# Multiparameter Continuous Physiological Monitoring Technologies in Neonates Among Health Care Providers and Caregivers at a Private Tertiary Hospital in Nairobi, Kenya: Feasibility, Usability, and Acceptability Study

**DOI:** 10.2196/29755

**Published:** 2021-10-28

**Authors:** Amy Sarah Ginsburg, Mai-Lei Woo Kinshella, Violet Naanyu, Jessica Rigg, Dorothy Chomba, Jesse Coleman, Bella Hwang, Roseline Ochieng, J Mark Ansermino, William M Macharia

**Affiliations:** 1 UW Clinical Trial Center University of Washington Seattle, WA United States; 2 Department of Obstetrics and Gynecology British Columbia Children’s and Women’s Hospital and The University of British Columbia Vancouver, BC Canada; 3 Centre for International Child Health BC Children’s Hospital Vancouver, BC Canada; 4 School of Arts and Sciences Moi University Eldoret Kenya; 5 Department of Anesthesiology The University of British Columbia Vancouver, BC Canada; 6 Department of Pediatrics Aga Khan University Nairobi Kenya; 7 Evaluation of Technologies for Neonates in Africa Nairobi Kenya

**Keywords:** infants, Africa, medical technology design, user perspectives, in-depth interviews, direct observations

## Abstract

**Background:**

Continuous physiological monitoring technologies are important for strengthening hospital care for neonates, particularly in resource-constrained settings, and understanding user perspectives is critical for informing medical technology design, development, and optimization.

**Objective:**

This study aims to assess the feasibility, usability, and acceptability of 2 noninvasive, multiparameter, continuous physiological monitoring technologies for use in neonates in an African health care setting.

**Methods:**

We assessed 2 investigational technologies from EarlySense and Sibel, compared with the reference Masimo Rad-97 technology through in-depth interviews and direct observations. A purposive sample of health care administrators, health care providers, and caregivers at Aga Khan University Hospital, a tertiary, private hospital in Nairobi, Kenya, were included. Data were analyzed using a thematic approach in NVivo 12 software.

**Results:**

Between July and August 2020, we interviewed 12 health care providers, 5 health care administrators, and 10 caregivers and observed the monitoring of 12 neonates. Staffing and maintenance of training in neonatal units are important feasibility considerations, and simple training requirements support the feasibility of the investigational technologies. Key usability characteristics included ease of use, wireless features, and reduced number of attachments connecting the neonate to the monitoring technology, which health care providers considered to increase the efficiency of care. The main factors supporting acceptability included caregiver-highlighted perceptions of neonate comfort and health care respondent technology familiarity. Concerns about the side effects of wireless connections, electromagnetic fields, and mistrust of unfamiliar technologies have emerged as possible acceptability barriers to investigational technologies.

**Conclusions:**

Overall, respondents considered the investigational technologies feasible, usable, and acceptable for the care of neonates at this health care facility. Our findings highlight the potential of different multiparameter continuous physiological monitoring technologies for use in different neonatal care settings. Simple and user-friendly technologies may help to bridge gaps in current care where there are many neonates; however, challenges in maintaining training and ensuring feasibility within resource-constrained health care settings warrant further research.

**International Registered Report Identifier (IRRID):**

RR2-10.1136/bmjopen-2019-035184

## Introduction

Globally, neonatal mortality remains persistently high, with a disproportionate burden in Sub-Saharan Africa [1]. Technologies that allow for early detection of neonatal physiological instability and help guide appropriate interventions have the potential to reduce morbidity and mortality [2]. In resource-constrained health care settings where staffing shortages of trained health care providers (HCPs) may compromise capacities for adequate monitoring and management, such technologies may prove life-saving [2].

The Evaluation of Technologies for Neonates in Africa (ETNA) project was conceived with the goal of advancing and supporting development, as well as evaluation of technologies for use in neonates in resource-constrained settings. The project seeks to boost the development and optimization of promising neonatal diagnostic and care technologies that could be applied in resource-constrained settings by establishing an Africa-based evaluation platform. This is achieved through global collaboration with partners with expertise in medical technology development and evaluation, as well as neonatal and child health. Critical to medical technology design, development, deployment, and eventual uptake and acceptability is understanding user perspectives in the intended setting. Evidence of the feasibility, appropriateness, and acceptability of innovative approaches for improving maternal and neonatal health has not been adequately investigated, which has implications for scale-up [3]. We assessed the feasibility, usability, and acceptability of 2 existing noninvasive, multiparameter, continuous physiological monitoring (MCPM) technologies developed by technology developers EarlySense and Sibel for use in neonates in an African health care setting.

## Methods

### Study Design

**Figure 1:**
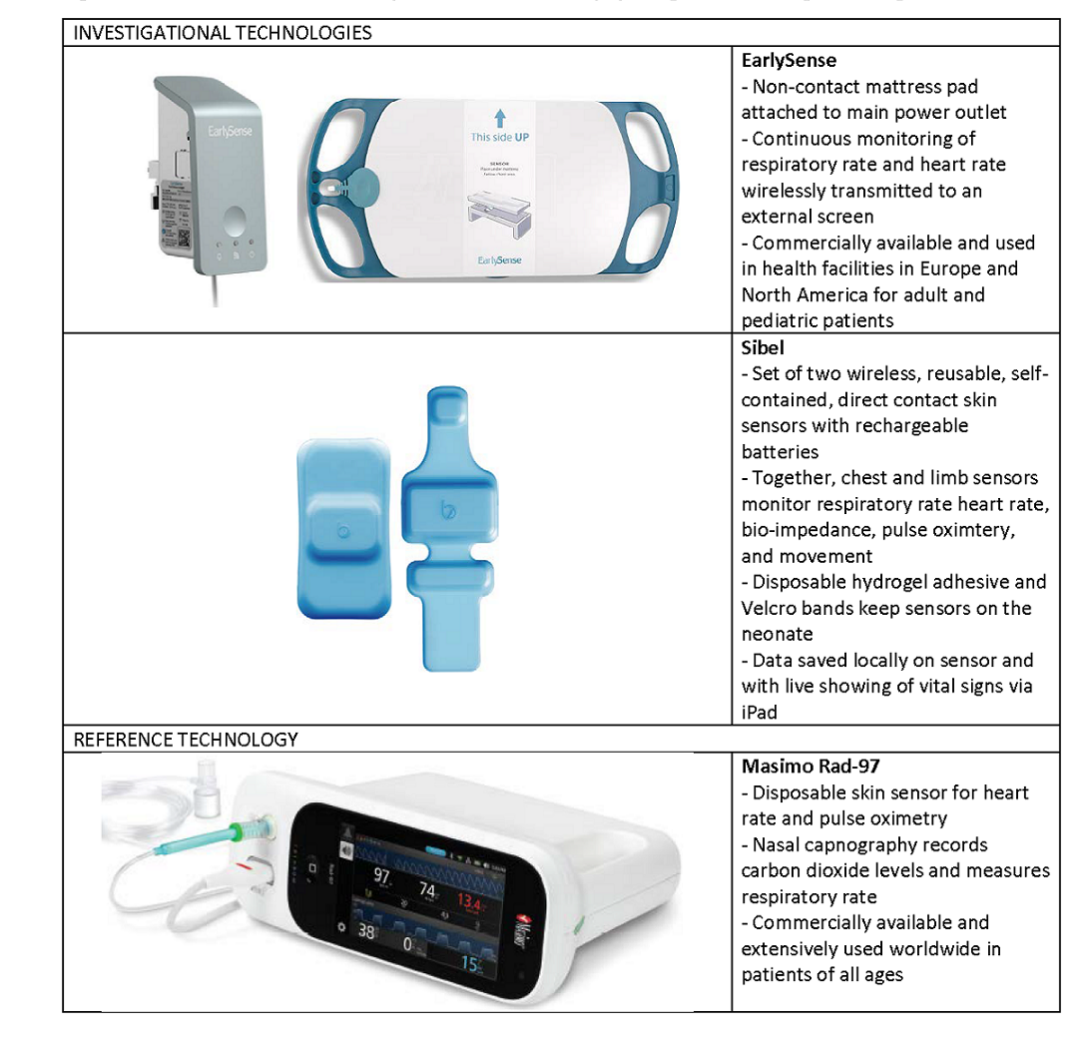
Overview of the 3 multiparameter continuous physiological monitoring technologies.

We conducted a qualitative study comprising in-depth interviews and direct observations using a cross-sectional design. This substudy was part of the larger ETNA project to evaluate the accuracy, reliability, and performance of 2 investigational noninvasive MCPM technologies in neonates when compared with verified reference technologies (Figure 1) [4]. The qualitative component used a descriptive and interpretive approach to understand the meanings respondents ascribed to feasibility, usability, and acceptability [5]. Feasibility comprises systemic factors, including hospital infrastructure and operational capacities, as well as functional capacities of the HCP available [6]. Usability comprises design factors affecting user experience, including features that support or hinder the operation of the technology for its intended purpose, such as ease of and efficiency in use and frequency of errors, memorability to a casual user, and user satisfaction with the system [6,7]. Acceptability comprises 2 dimensions: the willingness of HCPs to use the technology during patient interactions and the willingness of caregivers to have the technology used with their neonates [6].

The study is reported based on the *Consolidated Criteria for Reporting Qualitative Research* (COREQ; Multimedia Appendix 1) [8]. Ethics approvals were obtained from Western Institutional Review Board 20 191 102 (Puyallup, Washington), and the Aga Khan University Nairobi Research Ethics Committee 2019/REC-02 (v2; Nairobi, Kenya).

### Study Setting

The study was conducted at the Aga Khan University Hospital, Nairobi, a tertiary teaching and referral hospital with neonatal intensive care and high dependency units. The Aga Khan University Hospital is the busiest private hospital maternity unit in Nairobi, delivering approximately 4,500 neonates a year, and serves as a tertiary referral center for Kenya as well as the East Africa region. The neonatal high dependency unit has an 8-bed capacity and admits, on average, 5 to 10 neonates per week, with an approximate nurse-to-neonate ratio of 1:3. The ETNA project worked within the neonatal high-dependency unit and employed 2 research nurses to support the study.

### Recruitment and Selection

A purposive study sample was drawn to include a wide variety of perspectives on the feasibility, usability, and acceptability of the 3 MCPM technologies. The sample consisted of health care administrators, including hospital leadership and administrative staff involved in the procurement of neonatal hospital equipment, HCPs who were direct users of the MCPM technologies (health care provider—direct [HCP-Ds]), indirect HCPs involved in neonatal care (HCP-Is), and caregivers. A sample size of 12 HCPs and 5 health care administrators was estimated to cover perspectives from the staffing positions available and selected from a predefined list of current hospital staff generated by the ETNA team. HCP-D were recruited from ETNA nursing staff. HCP-D was trained for the study and had experience working with the technologies, whereas HCP-I (facility-based neonatal consultants, pediatric residents, and nurses) were oriented to the technologies during the interviews. A sample size of 10 caregivers, including mothers and fathers of neonates enrolled in the ETNA study, was estimated to reach data saturation because multiple technologies were used with each neonate during their hospital stay.

Study recruitment was publicized using flyers, and study participants were approached in person by a member of the qualitative study team, who were hired as part of the substudy and did not know the participants before the study. Interviewers first introduced themselves as members of the ETNA study team and explained the study in detail.

### Data Collection

In-depth interviews with health care administrators, HCPs, and caregivers and direct observations were conducted between July and August 2020. A Kenyan research consultant (VN, PhD in sociology, female) and a research assistant (Diploma in health sciences, female) were hired by the ETNA substudy to collect data. The research assistant underwent a 3-day intensive training in qualitative research methods led by VN before conducting the interviews.

The semistructured interview guide and observation guide (Multimedia Appendices 2 and 3) were piloted within the Kenyan data collection team during training to refine the questions. Face-to-face interviews were conducted in a private place within the hospital after the study participants provided written informed consent. The 30- to 45-minute interview was conducted in English or Kiswahili, the major local language in Kenya, depending on participant preference. One participant opted for a mix of Kiswahili and English, whereas the rest of the participants opted for English. Observations were conducted after obtaining written informed consent from HCP-D and followed a structured guide covering preparation and initial technology application, ongoing monitoring or troubleshooting, and technology disconnect, removal, and cleaning. Interviews were audio-recorded with permission, and data collectors took field notes while conducting the interviews. No repeat interviews were conducted.

### Data Analysis

Interviews were transcribed verbatim, translated into English as needed, and managed using NVivo 12 software (QSR International). We used a thematic approach to analyze the data following the methods described by Braun and Clarke to become familiar with the data, generating initial codes, collating identified codes into themes, and describing themes using illustrative quotes [9]. A coding framework was developed deductively from the study objectives to cover feasibility, usability, acceptability, and emergent themes from the transcripts. The coding framework was developed in consensus between the ETNA study team (ASG, MWK, VN, JR, DC, JC, and WMM), and VN conducted the primary coding with review by MWK (Multimedia Appendix 4). Confidentiality was maintained by limiting access of study materials to authorized personnel and ensuring that no identifying information was included in the analysis.

## Results

### Overview

Overall, the use of the relevant technologies was observed with 12 neonates, and observations took between 2 and 10 minutes per technology. In addition, 27 interviews were conducted, including 10 caregivers (9 mothers, 1 father), 2 HCP-D (study nurses), 10 HCP-I (4 medical doctors, 6 nurses), and 5 health care administrators (nurse managers, program administrators, and hospital unit supervisors). One HCP-I and no caregiver declined to participate. All health care administrators and HCPs had a postsecondary education. There were 2 HCPs with diplomas in nursing (1 HCP-D and 1 HCP-I), and all other health professionals had bachelor’s or master’s degrees. Health professionals had a median of 9 (<1-29) years of work experience in the medical field. In addition, all but 1 caregiver had a postsecondary education. The median age of caregivers was 33 (range 28-38) years, and they had a median of 2 (range 1-3) children. Caregivers were largely employed in professional occupations, including nursing, banking, human resource services, travel consultancy, business, sales, civil service, and farming.

### Feasibility Factors for the Investigational Technologies

Health care administrators described challenges in staffing and maintenance of training in neonatal units as a key feasibility consideration for the development of neonatal MCPM technologies. A health care administrator described:

I am finding it difficult to get the expertise that we require because...we don’t have many institutions who are training for critical care...neonatal nursing...[P]eople are learning on the job.

Another health care administrator highlighted:

There has been a lot of turnover in the newborn unit. So...you need to now make sure that you are training...on a continuous basis. It is not just about the equipment; the staff also need to have a very good understanding of how that equipment function.

Within the context of high staff turnover and on-the-job training, simple MCPM technologies were valued for the minimal training required and ease of application.

The minimal training required was a major facilitator reported for feasibility by the participants. Most HCPs and health care administrators (11/17, 65%) reported that the investigational technologies appeared to be easy to train for use and built on existing clinical skills. Referencing the EarlySense technology, an HCP-D nurse highlighted:

You only need very minimal training… just [place] it under the mattress and it monitors the baby, monitors the pressure. Very easy to use.

An HCP-I nurse noted that the Sibel technology could be easily learned within a few hours mentored by a current user:

[it requires] like an on-job training, like maybe a few hours, because...it is not...totally new from what is being used.

Feasibility challenges reported included the requirement of ancillary equipment, Wi-Fi requirements, and concerns about integration with existing facility equipment.

A minority of HCP and health care administrators expressed concerns about the requirements for external screens (2/17, 12%) and Wi-Fi (6/ 17, 35%). Equipment integration concerns were expressed particularly among health care administrators (3/5, 60%). A health care administrator said:

...we would want all the information in one place, and not this one here and the other on the other side...so that clinicians are not looking for information in two or three different places...

Affordability has also been raised as an important issue for feasibility. An HCP-I nurse said of the EarlySense technology:

Number one thing will be the cost...If the cost is higher, then [the hospital administrators] will have to weigh which is a cheaper option that will give more or less the same results.

Because the investigational MCPM technologies were perceived as simple, there was an expectation that costs would be affordable. A health care administrator said of the Sibel technology:

They should be about 10,000 shillings (approximately 90 USD) and not more than that. They are basic equipment.

### Usability Factors for the Investigational Technologies

Ease of use and efficiency of the technologies for neonatal care were major usability facilitators reported by most HCPs (9/12, 75%), health care administrators (4/5, 80%), and caregivers (7/10, 70%). An HCP-I nurse noted the EarlySense technology “is quite simple than our normal standard monitoring device here...it looks easier to use.” Speaking about the Sibel technology, an HCP-D nurse said:

It even [has] more functions than our current cardiac monitor so that’s a plus that we are having less manipulation to the baby in terms of attachment, but we are having much results. You can see more...heart rate, respirations, we can see movement, we can see temperature...

Ease of use was also reflected in observations; trained HCP-D nurses were able to prepare and initiate the technologies, monitor, and disconnect smoothly without many errors or assistance from other HCPs. Caregivers shared that they found the investigational technologies easy to understand and memorable even as casual users while monitoring their neonate at the hospital. Regarding the EarlySense technology, a mother said:

At a glance, you're able to know all your readings...You're actually able to monitor at a glance; you don't need to worry.

Another mother said of the Sibel technology:

...*...on her iPad, I could see the oxygen [levels]. It was on the right levels.*

The potential for wireless features to improve work efficiency was another major usability factor reported by the participants. Approximately half of the caregivers, HCPs, and health care administrators (13/27, 48%) commented that the potential of the wireless or noncontact features of the investigational technologies to transmit information to an external screen and remotely monitor multiple neonates could increase the speed of HCP detection and reaction to changes in vital signs. An HCP-D nurse commented on the EarlySense technology:

The screen that you are using to display the results, you can put it at the nursing station...So you can monitor many babies at the same time. It will reduce workload...

Speaking of the Sibel technology, a mother said:

You could be in a facility where babies are so many, so the service provider, they're overwhelmed. But if there are such devices that they're able to relay information faster, that means so many babies at least can be observed comfortably, so you save lives.

Fewer attachments have also been reported to increase efficiency in care by reducing the need to disconnect and reconnect the neonate during HCP examinations and caregiving needs, such as breastfeeding. Speaking of the EarlySense technology, a health care administrator said:

...the coding is remote. It doesn't interfere with routine care...Like sometimes, I have to remove certain wires to be able to examine a baby properly. So, the fact that it leaves the baby unencumbered with all those things I think is a huge advantage

However, with the EarlySense technology specifically, there were concerns from HCP and health care administrators (5/17, 29%) of monitoring during disruptions when the neonate was off the mattress during breastfeeding or otherwise being carried by caregivers.

Small size and portability were reported by some HCPs and health care administrators (8/17, 47%) as potential facilitators and challenges to usability. Although portability and convenience are linked, there was also concern about misplacement and theft because of their small size and portability. For example, a health care administrator said that it would be critical for the Sibel technology:

...to make sure that these things aren’t lost by staff...We might buy them, but at the end of the year, they might all be lost. Because, you know something which is attached and something which is a little bit big might be better.

Other usability challenges included concerns about infection control, shared by 41% (7/17) of the HCPs and health care administrators. In addition, 26% (7/27) of overall participants expressed concerns that equipment may be too large for preterm and low-birth-weight neonates. The plastic material of the Sibel technology was deemed easy to clean, but there was a preference for disposable items to streamline infection control processes at the hospital. Equipment that is too large for preterm and low-birth-weight neonates may lead to potentially poor application and inaccurate readings. For example, regarding concerns that the sensors for the Sibel technology would not fit a preterm neonate, a health care administrator shared:

For the baby’s chest, some of them like the preterms...I don’t know whether it would be so big, and then if it is big, then it is not connecting well.

### Acceptability Factors for the Investigational Technologies

Perceptions of neonate comfort were a major acceptability facilitator reported by many of the HCPs (6/ 12, 50%), health care administrators (4/5, 80%), and caregivers (610, 60%). The investigational technologies were seen as more comfortable and did not interfere with the neonates’ movements and sleep. Speaking about the EarlySense technology, one mother said:

It doesn’t interfere in any way with the baby. The baby is sleeping; they can just sleep, you know?...It’s painless while it’s measuring.

Another mother said, of the Sibel technology:

...*the baby didn’t seem uncomfortable...it didn’t cause the baby any discomfort.*

Half of the caregivers interviewed also mentioned that the investigational technologies were simpler, less intimidating, and more acceptable than conventional monitoring technologies.

Concern about potential harm around electrical fields and wireless connectivity was a major acceptability challenge mentioned by caregivers (8/10, 80%) and HCPs (7/12, 58%). For example, as one mother said, about the EarlySense technology:

...okay, they’re not using wires, so what are they using? Is there radiation, you know, that can harm my child?...of course, we asked about that, and we were told no, they’re safe...The concerns...were put to rest.

Speaking about the Sibel technology, an HCP-I nurse highlighted concerns shared by both caregivers and herself:

Now, you are not seeing any...wires moving from that device to...the screen next to the baby, so they [parents] want to know how that information is being passed...I am also wondering how it is working with that Bluetooth thing...so...personally I will go with the old version.

### Comparisons With the Reference Technology

A few HCPs and health care administrators (3/17, 18%) shared that in comparison with the investigational technologies, the complexity of the Masimo Rad-97 reference technology may require longer training. However, in contrast to the requirement of external screens or Wi-Fi with the investigational technologies, some HCPs and health care administrators shared that the reference technology is feasible within the Kenyan context because it is a stand-alone unit (3/17, 18%) and does not require Wi-Fi (2/17, 12%). For example, a health care administrator said:

...you don’t necessarily need another device to monitor, unlike Sibel where you need a [tablet]...In terms of feasibility, I would go for the implementation of Rad-97 first...

In addition, an HCP-I nurse said:

Aah, what I like about it is that...I don’t think it requires those WiFi things...so it can be used anywhere, any part of the country.

Of the 27 respondents, 4 (15%) mentioned that similar to the investigational technologies, the Masimo Rad-97 technology would be useful for care; 35% (6/17) of HCP and health care administrators shared that the Masimo Rad-97 technology seemed to have most of the features of the larger, wall-mounted conventional monitoring technology, but in a small and portable design.

In contrast to the investigational technologies, perceptions of neonate discomfort negatively impacted the acceptability of the reference technology, particularly with the nasal prong for capnography (12/ 27, 44%). Respondents (11/27, 41%) stated that wires and other attachments represent critical care and embody the seriousness of the health condition. A father shared:

Sometimes too many wires tend to shock...You might feel that the baby is in danger...Because the wires reflect the baby is, uh, is in dire need of help.

The nasal prong was seen as part of oxygen delivery, which was especially stressful for caregivers with neonates previously in intensive care. A mother shared:

I freaked out because having been in the same situation for the past one week...the first question that came into my mind is the baby going to struggle again breathing using those tubing on the nose.

An HCP-D nurse shared that caregivers were sometimes reluctant to accept the reference technology, as they perceived the nasal prong as invasive.

The facilitator of acceptability for the reference technology was brand familiarity, shared by HCPs (5/12, 42%) and health care administrators (3/ 5, 60%). An HCP-I physician described:

It is a device that has been used in the past, and its still being used all over the world. It’s a no brainer. It’s like going and asking someone “should you drive a Mercedes” it’s a known brand.

Feasibility, usability, and acceptability factors for the investigational and reference technologies included a myriad of facilitators and barriers (Table 1; Multimedia Appendices 5 and 6). Some factors have been reported to be potential facilitators and barriers.

**Table 1:** Feasibility, usability, and acceptability factors.

Factors	Investigational technologies			Reference technology	
	Facilitators	Barriers	Facilitators		Barriers
Feasibility	Easy to train for use	Requirement of ancillary equipment Wireless connectivity requirements (Sibel Bluetooth or EarlySense Wi-Fi) Integration capacities with existing equipment	Stand-alone unit Does not require Wi-Fi		Longer training because of complexity
Usability	Ease of use and useful for care Wireless features improve work efficiency Ability for infection control Small size and portability	Monitoring disruptions; such as when neonates are restless neonates or off mattress (EarlySense) Need for appropriate preterm and low birthweight sizing Ability for infection control Small size and portability	Useful for care Small size and portability		Small size and portability
Acceptability	Perceptions of neonate comfort Simple and less intimidating	Concerns about side effects from wireless connections	Brand familiarity		Perceptions of neonate discomfort because of nasal capnography Wiring and tubing linked to critical care

### Use Cases

HCPs and health care administrators shared that the investigational and reference technologies would be useful in different components of neonatal care at the hospital. As one health care administrator said, “I think in their own different capacity, they all have potential.”

Investigational technologies were especially recommended in the postnatal ward or nursery where healthy preterm and low-birth-weight neonates were being monitored. In these areas, there may be many neonates, largely under the care of their mothers and in stable health. Respondents suggested that the EarlySense technology would be useful “for babies who are being monitored [but] who are not so badly off” (HCP-I nurse), for “the postnatal babies...[where] there are many babies being continuously monitored” (HCP-D nurse), and for “a baby who we didn’t expect any sepsis or any challenges, and from this, we [would] be able to capture early signs of infection” (health care administrator). A health care administrator recommended the Sibel technology for the postnatal ward:

A mother who has delivered and has her baby needs to rest, and yet we have to monitor that baby. I would want that baby to be put on this. She can breastfeed and whatever she is doing, I can still be able to see the patterns and trends.

The reference technology was described as more suitable for neonates requiring more critical care, where neonates may be largely under the care of an HCP rather than caregivers. *“Especially with capnography in place,”* an HCP-D nurse said that the reference technology is not appropriate “for the postnatal babies because it makes [parents] feel like their baby is very sick or maybe on oxygen.” She also said:

I don’t think it is appropriate for postnatal, the wiring and the limited space that is there...and...because the baby has to be unplugged from the wires for breastfeeding or bathing, so it is not exactly feasible where neonate is under almost complete care of the mother.

However, she said that:

for babies in HDU or newborn ICU, it is very much feasible, as...it is familiar to what we are using.

## Discussion

### Principal Findings

The purpose of this qualitative study was to assess the feasibility, usability, and acceptability of 2 noninvasive MCPM technologies for neonates in an African health care setting. Study participants reported that the investigational technologies were feasible and useful in the care of neonates at the Aga Khan University Hospital in Nairobi. Feasibility facilitators included simple training requirements, whereas infrastructural requirements such as Wi-Fi, external display screens, and limited integration with existing equipment were reported as potential barriers. Usability facilitators included ease of use and wireless features, and concerns of possible harm from wireless connections and mistrust of unfamiliar technologies emerged as potential barriers to acceptability. Appropriate sizing for preterm and low-birth-weight infants, portability, human resource requirements and training, and perceptions of wireless technologies were identified as key issues to consider during the development and implementation of neonatal MCPM technologies.

### Implications for Practice

Our experience with MCPM technologies highlights the potential of different technologies for different neonatal care settings. The EarlySense technology was recommended for the postnatal ward where neonates were largely in stable health conditions and there were more neonates than nurses who could regularly monitor. Because the EarlySense technology only monitored while the neonate was resting on the mattress, the Sibel technology may be more appropriate during kangaroo mother care when the neonate spends most of their time on the caregiver’s chest. The investigational technologies’ ease of use supported their function within less critical areas of neonatal care, where the neonate is largely under the care of family members. In contrast, the reference technology used in this study was valued as a more compact and portable version of the larger, wall-mounted existing monitoring systems in intensive care units. Contextually appropriate MCPM technologies are particularly needed for the management of clinically unstable neonates to support early and safe initiation of evidence-based interventions such as kangaroo mother care and to monitor emerging complications such as hypothermia during bubble continuous positive airway pressure for respiratory distress [10,11]. This is important because immediate kangaroo mother care of low-birth-weight infants in critical condition has been shown to reduce infant mortality rates compared with conventional kangaroo mother care initiated after stabilization [12].

Although wireless features of the investigational technologies supported usability and acceptability in certain dimensions, including the potential for remote monitoring, simple design, less interference with care, increased comfort, and concern for potential health risks with wireless connectivity emerged as an unexpected theme. A study evaluating the acceptability of a wireless fetal heart rate monitoring device among pregnant women in rural Uganda also reported concerns among mothers about possible negative effects of electromagnetic radiation [13]. An improved understanding of barriers to and enablers of innovative neonatal health technologies for resource-constrained settings is a recognized gap in the literature [3]. Two reviews of wearable continuous monitoring sensors for neonates compiled products and key features but did not investigate acceptability or implementation factors [14,15]. Concerns about potential side effects from wireless connections and electromagnetic fields emphasize the importance of caregiver engagement and the need to work with HCPs to address clients’ mistrust of and fear of novel technologies. Caregivers expressed fear because of a lack of understanding of these technologies, but the fear appeared to be alleviated with HCP explanation for some.

Study findings such as identifying use case scenarios for different neonatal MCPM technologies and fears that wireless technologies may have adverse health effects highlight the importance of evaluating feasibility, usability, and acceptability during the development of medical technologies. Although medical technologies may demonstrate efficacy, their adoption, uptake, and use may be limited if implementation factors are not considered and incorporated during technology development. The potential impact of innovative neonatal MCPM technologies is substantial, particularly in resource-constrained settings. Frequently, there may be little to no neonatal continuous monitoring available in these settings, despite being routine in high-income settings for those who require it. This lack of monitoring may contribute to the higher rates of neonatal morbidity and mortality in resource-constrained settings [16-18]. An observational study at Kenyatta National Hospital in Nairobi reported that very few neonates had their vital signs recorded in the first hour of life, and more than half did not receive a temperature recording (54%), heart rate recording (56%), or respiratory rate recording (56%) on the first day of hospital admission [16]. Observations at 6 hospitals in Nairobi County found that missed vital sign monitoring and other nursing tasks were associated with nursing shortages and high patient workloads [17]. MCPM technologies are valuable for improving the quality of neonatal care by expanding nurses’ capacities to monitor more neonates regularly and efficiently.

### Strengths and Limitations

Of note, this qualitative study was conducted at a private, tertiary hospital where the study participants were highly educated, and almost all of the caregivers interviewed had university education and professional employment. In addition, limiting the generalizability of our study findings is that private and public hospitals in Kenya have dramatically different nursing workloads and infrastructure, with median ratios of 3 infants to 1 nurse at private hospitals around Nairobi and 19 infants to 1 nurse at public hospitals [17]. With a reliable back-up electrical system and maintenance team on staff at Aga Khan University Hospital, Nairobi, electrical outages, technology malfunction, and maintenance were not highlighted as feasibility concerns by our study participants. The feasibility of these investigational MCPM technologies for neonates has important implications for the sustainability and prevention of technology graveyards of nonfunctional or locally inappropriate technologies. Future research can explore whether feasibility, usability, and acceptability issues shift in a public hospital setting where resources may be more constrained. Another limitation of the study is that usability was not directly assessed among nontrained (HCP-I) users. The strengths of the study include the use of direct observations to support interview findings, as well as conducting in-depth interviews with caregivers, HCPs, and health care administrators to understand a diversity of perspectives.

### Conclusions

MCPM for neonates is a critical component of comprehensive care that supports the effectiveness of other neonatal interventions. Our study examined the feasibility, usability, and acceptability of 2 investigational MCPM technologies for neonates compared with a reference MCPM technology and found that the different technologies fit different areas within the continuum of neonatal care at the hospital. Although each technology presented advantages suited for different neonatal care domains, challenges in maintaining training and ensuring feasibility within resource-constrained health care settings warrant further research.

## Appendix

Multimedia Appendix 1 COREQ (Consolidated Criteria for Reporting Qualitative Research) Checklist.

Multimedia Appendix 2 In-depth interview guides.

Multimedia Appendix 3 Direct observation guide.

Multimedia Appendix 4 Coding tree.

Multimedia Appendix 5 Feasibility, usability, and acceptability factors of investigational and reference technologies with illustrative quotes.

Multimedia Appendix 6 Themes by technology and participant group.

## Abbreviations

COREQ: Consolidated Criteria for Reporting Qualitative Research
ETNA: Evaluation of Technologies for Neonates in Africa
HCP: health care provider
HCP-D: health care providers-direct users of the MCPM technologies
HCP-I: health care providers-indirect users of the MCPM technologies
MCPM: multiparameter continuous physiological monitoring
