# Supplementary material for: Multiparameter Continuous Physiological Monitoring Technologies in Neonates Among Health Care Providers and Caregivers at a Private Tertiary Hospital in Nairobi, Kenya: Feasibility, Usability, and Acceptability Study
Source: J Med Internet Res. 2021 Oct 28;23(10):e29755. doi: 10.2196/29755 (PMC8587184; doi:10.2196/29755)
Supplement: Multimedia Appendix 2 [file jmir_v23i10e29755_app2.pdf]

S2 File ETNA Qualitative Study Interview Guides

2.1 In-Depth Interview Guide – Caregiver

**Administrative information**	
**Caregiver ID number:**	**Sex:** ☐ Female ☐ Male
**Date caregiver informed consent form (ICF) signed:** |D|D| - |M|M|M| - |Y|Y|Y|Y|	
**Caregiver ICF signed prior to any study questions?** ☐ Yes ☐ No	
**Name of research staff who explained the ICF:**	
**Does the caregiver agree to be audio recorded?** ☐ Yes ☐ No<br>If Yes, was the interview audio recorded? ☐ Yes ☐ No<br>If No, why was the interview not recorded? ____________________	
**Name of interviewer:**	
**Date of interview:** |D|D| - |M|M|M| - |Y|Y|Y|Y|	
**Location of interview:**<br>☐ **Aga Khan University – Nairobi Hospital**<br>☐ **Pumwani Maternity Hospital**	
**Interview start time:** |H|H| : |M|M| *military time*	

<u>Instructions for qualitative research staff</u>:

Use this document as a guide to conduct the interviews with the **caregivers**.

Conduct the interview in the language with which the caregiver feels most comfortable.

The interview should take place in a quiet place that allows privacy.

Please introduce each question separately. The interview must flow as a conversation. If you notice that the caregiver is hesitant in answering, does not give an in-depth response, or the response is not satisfactory, please probe or ask follow-up questions, but do NOT prompt any specific answer. Several probes are suggested, and you may also ask follow-up questions that are not listed in this guide but are necessary for the complete expression of the caregiver's views.

Please **record the interview** using the audio recorder (if caregiver consent is provided) and state the ETNA Caregiver ID number.

All comments from the caregiver should be recorded/written verbatim.

Please cross-check the narratives written with the recorded version as a reference and correct as necessary.

All responses must be kept confidential. Do not discuss or share responses with anyone outside of the ETNA study team.

Script to initiate the interview

"Hello, my name is __________ and I am a researcher with the ETNA project and we are evaluating monitoring devices for newborns. We want to hear about your experiences and learn from your thoughts and feelings. We will keep what you tell us today confidential, which means that nothing you say will be directly linked to you so please feel free to share. If you feel uncomfortable with any questions, let me know and we will skip it. Before we start, do you have any questions for me? Is it ok to begin? Thank you, I will start the audio-recording now."

A. Demographic information

1. First, we will start with some questions about yourself, what is your age?
2. Did you attend any schooling? If so, what class (level) did you complete?
3. Would you be able to tell us a little about yourself and what you/your husband do for a living?
4. Where do you and your family live? How far away is it from this hospital?
5. How many people live together in your house and what is their relationship to you?
6. How many children do you have? What is your role in caring for your newborn?

B. Birth history, pregnancy and healthcare facility experience

If this some of this information could be abstracted from patient hospital records, could consider using these records as a resource BEFORE the interview. Otherwise, please ask these questions.

1. How many pregnancies have you (or your wife, daughter-in-law, daughter) had? How many live births?
2. We would like to learn more about your experience with your most recent pregnancy. Could you tell us if you had any issues or complications during the pregnancy, labor or delivery?

Probes: What were your symptoms during pregnancy, length of labor, mode of delivery, how long admitted to the hospital?

3. Was your baby born early? If yes, do you know how early? (Another way to phrase this is "When were you expecting the baby and was the baby born earlier?")
4. Did your newborn have any health issues when he/she was born? If yes, what were they?

Probes: Examples include low birth weight (kangaroo mother care), infection at time of birth, birth defect, respiratory distress (trouble breathing), neonatal jaundice (put under the blue light), inability to breastfeed, etc.

5. What healthcare services did you and your newborn receive here at the hospital?
6. Are you happy with the quality of care you and your newborn received at this hospital? Could you explain with an example? What do you think could make the quality of care at this hospital better?

Probes: How did the staff treat you and your family? Did they seem trained/knowledgeable? Did they have enough equipment/supplies to care for you and your newborn?

7. How did you get to this hospital and how long did it take you to get here from your home? Why did you and your family decide to come to this hospital for delivery (or newborn care depending on their narrative)?
8. What other health facilities do you usually go to when you or your family needs medical care? When do you go to those other health facilities instead of this hospital?

C. Monitoring devices

1. What are your experiences with how healthcare providers monitor newborns receiving care at this hospital? How often do they come by to check your newborn and what do they usually check?
2. Do healthcare providers use any devices or technologies when they are doing a checkup on your newborn?
3. Are there any devices or machines that you are aware of that are used to monitor the newborn between checks by the healthcare provider?
4. Do you have any concerns about these devices? If so, could you explain with an example?

D. EarlySense InSight investigational device

Research staff shows EarlySense InSight device to the caregiver and explains how it works.

"The EarlySense InSight device is a contact-free newborn monitoring system. The system includes a sensor pad that is placed under the newborn's mattress to measure heart rate, breathing rate, motion, and sleep status. There is no physical contact between the newborn and the sensor pad. Information from the sensor pad is continuously transmitted to a monitor or tablet that can be read by hospital staff."

Allow them to touch the InSight device, the mattress pad and the cable that goes between them.

1. Have you ever seen this device before? Was this device used in the care of your newborn?
   If yes,
   a. What was the first thought that came to your mind when the healthcare provider told you about this device? How did you feel about this device being used for your newborn? How do you feel about it now? How did your husband (or family) react when they learned that your newborn was on this device?
   b. What did you like (if anything) about this device and how it was used? What did you dislike (if anything) about this device and how it was used? Please explain.
   c. Did the healthcare provider using the device run into any difficulties? What did they do?

   If no,
   a. Imagine if a doctor recommended using this device for your newborn, how would you feel? What do you think your husband (or family) would think if your newborn was put on this device?
2. Are there any problems you can think of with this device? Any concerns or parts/features you think might be harmful to newborns? In what situations? Please explain.
3. Do you think this device should be used in the care of newborns in this hospital? Why or why not?
4. Do you see any problems with using this device at this hospital? If so, could you explain with an example?
5. If there was a healthcare facility that used this device regularly to help care for newborns, would that make you want to go to that facility more or less? Why?

E. Sibel ANNE investigational device

Research staff shows Sibel ANNE device to caregiver and explains how it works.

"The Sibel ANNE device uses non-invasive sensors to continuously measure and record a newborn's heart rate, breathing rate, level of oxygen in the blood, and skin temperature. One sensor is attached to the newborn's chest and contains a battery. The second sensor is battery-free, ultra-thin, and is applied to the newborn's hand or foot. Information from the sensors is wirelessly transmitted to a monitor or tablet that can be read by hospital staff."

Allow them to touch the ANNE chest and limb sensors, the hydrogel, and iPad display fully.

1. Have you ever seen this device before? Was this device used in the care of your newborn?
   If yes,
   a. What was the first thought that came to your mind when the healthcare provider told you about this device? How did you feel about this device being used for your newborn? How do you feel about it now? How did your husband (or family) react when they learned that your newborn was on this device?
   b. What did you like (if anything) about this device and how it was used? What did you dislike (if anything) about this device and how it was used? Please explain.
   c. Did the healthcare provider using the device run into any difficulties? What did they do?

   If no,
   a. Imagine if a doctor recommended using this device for your newborn, how would you feel? What do you think your husband (or family) would think if your newborn was put on this device?
2. Are there any problems you can think of with this device? Any concerns or parts/features you think might be harmful to newborns? In what situations? Please explain.
3. Do you think this device should be used in the care of newborns in this hospital? Why or why not?
4. Do you see any problems with using this device at this hospital? If so, could you explain with an example?
5. If there was a healthcare facility that used this device regularly to help care for newborns, would that make you want to go to that facility more or less? Why

F. Masimo RAD-97 reference device
**Research staff shows Masimo Rad-97 device to caregiver and explains how it works.**<br>*"The Masimo Rad-97 is a non-invasive device that measures a newborn's heart rate, breathing rate, and level of oxygen in the blood. Information is collected through a skin sensor that is applied to the newborn's hand or foot and a tube that is inserted into the newborn's nostrils. The information is then continuously transmitted to a monitor that can be read by hospital staff."*<br>**Allow them to touch the Rad-97, skin sensor and capnography tube fully.**<br>1. Have you ever seen this device before? Was this device used in the care of your newborn?<br>**If yes,**<br>a. What was the first thought that came to your mind when the healthcare provider told you about this device? How did you feel about this device being used for your newborn? How do you feel about it now? How did your husband (or family) react when they learned that your newborn was on this device?<br>b. What did you like (if anything) about this device and how it was used? What did you dislike (if anything) about this device and how it was used? Please explain.<br>c. Did the healthcare provider using the device run into any difficulties? What did they do?<br>**If no,**<br>a. Imagine if a doctor recommended using this device for your newborn, how would you feel? What do you think your husband (or family) would think if your newborn was put on this device?<br>2. Are there any problems you can think of with this device? Any concerns or parts/features you think might be harmful to newborns? In what situations? Please explain.<br>3. Do you think this device should be used in the care of newborns in this hospital? Why or why not?<br>4. Do you see any problems with using this device at this hospital? If so, could you explain with an example?<br>5. If there was a healthcare facility that used this device regularly to help care for newborns, would that make you want to go to that facility more or less? Why

H. Closing
1. Taking into consideration the monitoring devices we have talked about today, could you rank the device (if any) you think is the best, second best and third choice in your opinion? Please explain why.<br>2. Do you have any other comments about any of the three devices that we did not talk about?<br>3. Do you have any other comments about newborn monitoring devices or any other comments or concerns overall that we did not get to talk about?
"Thank you for your time and the helpful information you have provided. Your feedback, along with feedback from other people we talk to will be used to recommend solutions for better care."
**Interview end time:** |H|H| : |M|M| *military time*

2.2 In-Depth Interview Guide – Healthcare Administrator (HCA)

Administrative information	
**HCA ID number:**	**Sex:** ☐ Female ☐ Male
**Date HCA informed consent form (ICF) signed:** |D|D| - |M|M|M| - |Y|Y|Y|Y|	
**HCA ICF signed prior to any study questions?** ☐ Yes ☐ No	

**Name of research staff who explained the ICF:**
**Does the HCA agree to be audio recorded?** ☐ Yes ☐ No<br>If Yes, was the interview audio recorded? ☐ Yes ☐ No<br>If No, why was the interview not recorded? ____
**Name of interviewer:**
**Date of interview:** |D|D| - |M|M|M| - |Y|Y|Y|Y|
**Location of interview:**<br>☐ **Aga Khan University – Nairobi Hospital**<br>☐ **Pumwani Maternity Hospital**<br>☐ **Other: ___________________________**
**Interview start time:** |H|H| : |M|M| *military time*

<u>Instructions for qualitative research staff</u>:

- Use this document as a guide to conduct the interviews with the **healthcare administrators (HCA)**.
- Conduct the interview in the language with which the HCA feels most comfortable.
- The interview should take place in a quiet place that allows privacy.
- Please introduce each question separately. The interview should flow as a conversation. If you notice that the HCA is hesitant in answering, does not give an in-depth response, or the response is not satisfactory, please probe or ask follow-up questions, but do NOT prompt any specific answer. Several probes are suggested, and you may also ask follow-up questions that are not listed in this guide but are necessary for the complete expression of the HCA's views.
- Please **record the interview** using the audio recorder (if HCA consent is provided) and state the ETNA HCA ID number.
- All comments from the HCA should be recorded/written verbatim.
- Please cross-check the narratives written with the recorded version as a reference and correct as necessary.
- All responses must be kept confidential. Do not discuss or share responses with anyone outside of the ETNA study team.

**Script to initiate the interview**
"Hello, my name is __________ and I am a researcher with the ETNA project and we are evaluating monitoring devices for newborns. We want to hear about your experiences and learn from your thoughts and feelings. We will keep what you tell us today confidential, which means that nothing you say will be directly linked to you so please feel free to share. If you feel uncomfortable with any questions, let me know and we will skip it. Before we start, do you have any questions for me? Is it ok to begin? Thank you, I will start the audio-recording now."

**G. Demographic information**
1. First, we will start with some questions about yourself, what is your age?<br>2. How many years of education and training have you received and what is your highest level of education completed? What is your medical background (e.g., doctor, nurse, technician, etc.)? Were you ever involved in patient care? In the care of newborns?<br>3. How long did you work in the medical field before working as a healthcare administrator? How long have you been working as a healthcare administrator?
**H. Healthcare administrator role**

1. What is your job title and current role here at this healthcare facility? How long (years, months if less than one year) have you been in the current position at this facility?
2. What are your responsibilities as a healthcare administrator at this facility?

Probes: What is your involvement (if any) in policy development for newborn care such as creating new protocols and/or adapting national guidelines? Please share what a typical day as a hospital administrator would be like for you.

I. Facility

1. What is the process of purchasing medical equipment at this healthcare facility?
   Probes: Who makes the decision to identify what medical equipment will be used in the hospital? Who makes the decisions on what to purchase? Are these decisions made on an individual hospital basis or decided at a local or national level by Ministry of Health?
2. What are the current constraints (if any) to providing care to newborns at this facility?
   Probes: What makes care more difficult? What would make it easier?
3. Does this facility have reliable access to electricity? When was the last electricity outage and how long do they typically last? What happens during power outages at your facility?
   Probes: How do power outages affect patient care? Is there a back-up power supply? If so, what is the process of using the backup power supply and are there any issues around its use (e.g., does it cover all of the equipment needed, any issues in getting permission for its use, fuel prices? Any voltage issues?)
4. Are you aware of any technologies that are being used in the delivery and newborn care wards at this facility, and if so, can you describe them? What are some concerns you have, or gaps in the technologies available, for maternal and newborn care at this facility?
   Probes: Which healthcare providers use the technologies? What technologies/brands are used? Do healthcare providers use any handheld or portable devices for maternal or newborn care (e.g., tablets or Smartphones)? If yes, please describe the technologies and their use.

J. Monitoring devices

We would especially like to learn about your perspectives on continuous monitoring devices.

1. Before this study, had you used continuous monitoring devices or seen them in use? Are you aware of any continuous monitoring devices being used at this healthcare facility outside of the ETNA study?
   Probes: If yes, where in the facility? For what purpose? How frequently are they used?
2. What do you think are some of the benefits of using continuous monitoring devices at your facility? What impacts do you think they have (if in current use) or would have (if not in current use) on routine care at this facility?
3. Do you have any concerns about using continuous monitoring devices? What are the challenges to using such devices at this facility? Are there any situations you think the use of monitoring devices would not be useful? If so, can you explain with an example?
   Probes: Tell me about how newborns are monitored in your facility? How is this different (if at all) for sick newborns?
4. What do you think would be needed to scale up the use of continuous monitoring devices at this facility? What enablers do you think could support this process?
5. Imagine if monitoring devices were scaled up at this facility, how do you think the nurses and doctors that work here would react? How do you think caregivers (mothers, parents, guardians, etc.) would react? What about outside stakeholders and decision-makers at local, county, and national levels?

K. EarlySense InSight investigational device

Research staff shows EarlySense InSight device to HCA and explains how it works.

"The EarlySense InSight device is a contact-free newborn monitoring system. The system includes a sensor pad that is placed under the newborn's mattress to measure heart rate, breathing rate, motion, and sleep status. There is no physical contact between the newborn and the sensor pad. Information from the sensor pad is continuously transmitted to a monitor or tablet that can be read by hospital staff. The system has been previously tested for safety in neonates."
Allow them to touch the InSight device, the mattress pad and the cable that goes between them.

6. Could you share what you like (if anything) about this device? What do you think would be useful in the care of newborns at your healthcare facility?
7. Could you share what you dislike (if anything) about this device? What about the device do you think could create difficulties in caring for newborns at your facility?
8. Do you think this device is suitable for use in your facility? What do you think would need to happen in order to successfully use this device in your facility?
   Probes: For example, staffing availability and skill to use the device, training, complexity of the device, availability of equipment and infrastructure needed for its use, durability and maintenance of device and components, access to spare parts, protocols and guidelines for use, counselling caregivers and informational materials? What could be the benefits/drawbacks?
9. How do you think healthcare providers, like doctors and nurses, would feel about this device?
10. How do you think caregivers, such as mothers, fathers, mothers-in-law and other family members, would feel about this device?
11. How do you think other healthcare administrators and decision-makers at local, county and national levels would react to a recommendation to implement this device at this facility
12. In your opinion, how much would your facility pay for a device like this? (Circle Response)

<$5000 KSh	$5000 – $10000 KSh	$10000 – $15000 KSh
$15000 - $20000 KSh	$20000 - $25000 KSh	>$25000 KSh

Please explain

L. Sibel ANNE investigational device

Research staff shows Sibel ANNE device to HCA and explains how it works.
"The Sibel ANNE device uses non-invasive sensors to continuously measure and record a newborn's heart rate, breathing rate, level of oxygen in the blood, and skin temperature. One sensor is attached to the newborn's chest and contains a battery. The second sensor is battery-free, ultra-thin, and is applied to the newborn's hand or foot. Information from the sensors is wirelessly transmitted to a monitor or tablet that can be read by hospital staff. The system has previously been tested for safety in neonates."
Allow them to touch the ANNE chest and limb sensors, the hydrogel, and iPad display fully.

1. Could you share what you like (if anything) about this device? What do you think would be useful in the care of newborns at your healthcare facility?
2. Could you share what you dislike (if anything) about this device? What about the device do you think could create difficulties in caring for newborns at your facility?
3. Do you think this device is suitable for use in your facility? What do you think would need to happen in order to successfully use this device in your facility?
   Probes: For example, staffing availability and skill to use the device, training, complexity of the device, availability of equipment and infrastructure needed for its use, durability and maintenance of device and components, access to spare parts, protocols and guidelines for use, counselling caregivers and informational materials? What could be the benefits/drawbacks?
4. How do you think healthcare providers, like doctors and nurses, would feel about this device?
5. How do you think caregivers, such as mothers, fathers, mothers-in-law and other family members, would feel about this device?

6. How do you think other healthcare administrators and decision-makers at local, county and national levels would react to a recommendation to implement this device at this facility?
7. In your opinion, how much would your facility pay for a device like this? (Circle Response)

<$5000 KSh	$5000 – $10000 KSh	$10000 – $15000 KSh	
$15000 - $20000 KSh	$20000 - $25000 KSh	>$25000 KSh	

Please explain

M. Masimo RAD-97 reference device

Research staff shows Masimo Rad-97 device to HCA and explains how it works.
"The Masimo Rad-97 is a non-invasive device that measures a newborn's heart rate, breathing rate, and level of oxygen in the blood. Information is collected through a skin sensor that is applied to the newborn's hand or foot and a tube that is inserted into the newborn's nostrils. The information is then continuously transmitted to a monitor that can be read by hospital staff. The system has previously been tested for safety in neonates."
Allow them to touch the Rad-97, skin sensor and capnography tube fully.

1. Could you share what you like (if anything) about this device? What do you think would be useful in the care of newborns at your healthcare facility?
2. Could you share what you dislike (if anything) about this device? What about the device do you think could create difficulties in caring for newborns at your facility?
3. Do you think this device is suitable for use in your facility? What do you think would need to happen in order to successfully use this device in your facility?
   Probes: For example, staffing availability and skill to use the device, training, complexity of the device, availability of equipment and infrastructure needed for its use, durability and maintenance of device and components, access to spare parts, protocols and guidelines for use, counselling caregivers and informational materials? What could be benefits/drawbacks?
4. How do you think healthcare providers, like doctors and nurses, would feel about this device?
5. How do you think caregivers, such as mothers, fathers, mothers-in-law and other family members, would feel about this device?
6. How do you think other healthcare administrators and decision-makers at local, county and national levels would react to a recommendation to implement this device at this facility?
7. In your opinion, how much would your facility pay for a device like this? (Circle Response)

<$5000 KSh	$5000 – $10000 KSh	$10000 – $15000 KSh
$15000 - $20000 KSh	$20000 - $25000 KSh	>$25000 KSh

Please explain.

H. Closing

1. Taking into consideration the three monitoring devices we have talked about today, can you rank the device (if any) you think is the best, second best and third choice in your opinion? Please explain why.
2. In terms of feasibility, which device (if any) do you think would be the most appropriate device for your healthcare facility and why?
3. In terms of acceptability, which device (if any) do you think healthcare providers would like the best and why? Which device (if any) do you think caregivers would prefer and why?
4. Do you have any other comments about any of the three devices that we did not talk about?
5. Do you have any other comments about newborn monitoring devices or any other comments or concerns overall that we did not get to talk about?

"Thank you for your time and the helpful information you have provided. Your feedback, along with feedback from other people we talk to will be used to recommend solutions for better care."

**Interview end time:** |H|H| : |M|M| *military time*

2.3 In-Depth Interview Guide – Healthcare Provider (HCP) Direct Use

**Administrative information**	
**HCP ID number:**	**Sex:** ☐ Female ☐ Male
**Date HCP informed consent form (ICF) signed:** |D|D| - |M|M|M| - |Y|Y|Y|Y|	
**HCP ICF signed prior to any study questions?** ☐ Yes ☐ No	
**Name of research staff who explained the ICF:**	
**Does the HCP agree to be audio recorded?** ☐ Yes ☐ No<br>If Yes, was the interview audio recorded? ☐ Yes ☐ No<br>If No, why was the interview not recorded? ____	
**Name of interviewer:**	
**Date of interview:** |D|D| - |M|M|M| - |Y|Y|Y|Y|	
**Location of interview:**<br>☐ **Aga Khan University – Nairobi Hospital**<br>☐ **Pumwani Maternity Hospital**	
**Interview start time:** |H|H| : |M|M| *military time*	

Instructions for qualitative research staff:

- Use this document as a guide to conduct the interviews with **healthcare providers (HCP) directly using the devices**.
- Conduct the interview in the language with which the HCP feels most comfortable.
- The interview should take place in a quiet place that allows privacy.
- Please introduce each question separately. The interview must flow as a conversation. If you notice that the HCP is hesitant in answering, does not give an in-depth response, or the response is not satisfactory, please probe or ask follow-up questions, but do NOT prompt any specific answer. Several probes are suggested, and you may also ask follow-up questions that are not listed in this guide but are necessary for the complete expression of the HCP's views.
- Please **record the interview** using the audio recorder (if HCP consent is provided) and state the ETNA HCP ID number.
- All comments from the HCP should be recorded/written verbatim.
- Please cross-check the narratives written with the recorded version as a reference, and correct as necessary.
- All responses must be kept confidential. Do not discuss or share responses with anyone outside of the ETNA study team.

**Script to initiate the interview**
"Hello, my name is ___________ and I am a researcher with the ETNA project and we are evaluating monitoring devices for newborns. We want to hear about your experiences and learn from your thoughts and feelings. We will keep what you tell us today confidential, which means that nothing you say will be directly linked to you so please feel free to share. If you feel uncomfortable with any questions, let me know and we will skip it. Before we start, do you have any questions for me? Is it ok to begin? Thank you, I will start the audio-recording now."

N. Demographic information
7. First, we will start with some questions about yourself, what is your age?<br>8. How many years of education and training have you completed and what is your highest level of education completed? What is your medical background/designation? (e.g., doctor, nurse, technician, etc.)<br>9. How long have you worked as 'doctor/nurse/technician/etc.?

O. Healthcare provider role
4. How long have you been employed at this healthcare facility?<br>5. What is your job title and current role here at this facility? How long have you been in this role at this facility?<br>6. What are your responsibilities in this role?<br>*Probes: Please share what a typical day as a healthcare provider would be like for you.*<br>*7.* Are you involved in patient care? If yes, please explain your patient care responsibilities.

P. Facility
5. What are the current constraints to providing care to newborns at this healthcare facility?<br>*Probes: What makes care more difficult? What would make it easier?*<br>6. How are newborns monitored at this facility? How is this different (if at all) for sick newborns?<br>7. Does this facility have reliable access to electricity? When was the last electricity outage and how long do they typically last? What happens during power outages at your facility?<br>*Probes: How do power outages affect patient care? Is there a back-up power supply? If so, what is the process of using the backup power supply and are there any issues around its use (e.g., does it cover all of the equipment needed, any issues in getting permission for its use, fuel prices? Any voltage issues?*<br>8. Do you have regular access to computers at this facility? If yes, do they work well?<br>*Probes: Do computers breakdown often? If yes, please describe how the computer breakdowns affect your work as a healthcare provider?*<br>9. Could you describe the technologies that are being used in the delivery and newborn care wards at this facility? What are some concerns you have, or gaps in the technologies available, for maternal and newborn care at this facility?<br>*Probes: Which healthcare providers use the technologies? What technologies/brands are used? Do healthcare providers use any handheld or portable devices for maternal or newborn care (e.g., tablets or Smartphones)? If yes, please describe the technologies and their use.*

Q. Monitoring devices
1. What is your role with the Evaluation of Technologies for Neonates in Africa (ETNA) research study? What are your ETNA-related responsibilities?<br>**We would especially like to learn about your perspectives on continuous monitoring devices.**<br>2. Before this study, had you used continuous monitoring devices or seen them used? Tell me about your experience with continuous monitoring devices.<br>*Probes: List devices used, then discuss each device sequentially (where used, for what purpose?). How frequently have you used these types of devices? Did you find them to be useful? If yes, how so? If no, why not? What sort of training did you receive for the use of these devices?*<br>3. Apart from the devices used in the ETNA study, are continuous monitoring devices used at this healthcare facility?<br>*Probes: If yes, where in the facility? For what purpose? How frequently do you use these devices?*<br>4. What do you think are some of the benefits of using continuous monitoring devices? What impacts do you think they could have on routine care at this facility?

5. Do you have any concerns about using continuous monitoring devices? What are the challenges to using such devices at this facility? Are there any situations you think the use of monitoring devices would not be useful? If so, could you explain with an example?
6. What do you think would be needed to scale up the use of continuous monitoring devices at this facility? What enablers do you think could support this process?
7. Imagine if continuous monitoring devices were scaled up at this facility, how do you think the nurses and doctors that work here would react? How do you think caregivers (mothers, parents, guardians, etc.) would react?

E. EarlySense InSight investigational device

The next set of question will focus on your experiences with the EarlySense InSight device.

Usability

13. Do you think that healthcare providers in this facility could develop the skills necessary to use this continuous monitoring device? Why or why not?
14. What sort of training did you receive on this device before you began using it?
Probes: Please describe length and method of training. Who provided training? Was training adequate? What additional training do you wish you had received? What sort of training do you think would be required for healthcare providers in this facility to use this device?
15. Which aspects of using this device were easy to learn? Which aspects were difficult?
Probes: Did using the device become easier or more difficult over time?
16. If you now feel comfortable using the device, how long did it take you to become comfortable? If not comfortable, why not?
17. What kind of support did you receive during this period? Please explain.
Probes: From device manufacturers, supervisors, coworkers, etc.?
18. What did you like (if anything) about this device overall? What did you dislike (if anything)? Are there any changes you would make to this device? If so, what are they?
Probes: For example, overall device setup/interface, ease of use, etc.? What about the different features: InSIght device, mattress pad, cable, mobile application, monitor screen/display, etc.?
19. Did this device make providing care to newborns at this facility easier or more difficult? How so?
Probes: For example, enable more care, interrupt care, etc.
20. Do you think the device would make care easier or more difficult if you could use the information collected and displayed by this device? How so?
21. Were there any questions you had about this device while you were using it? Please explain.
22. Did caregivers or other hospital staff ask you any questions about this device while you were using it? If so, what did they ask?
23. Are there situations where you think this device should not be used? If so, what are they?

Acceptability

24. How do you think other healthcare providers, like doctors and nurses, would feel about this device?
25. Based on your encounters with caregivers, such as mothers, fathers, mothers-in-law and other family members, how do you think they would feel about this device?
26. How do you think healthcare administrators and decision-makers at local, district and national levels would react to a recommendation to implement this device at this facility?
Probes: Discuss at each level (local, district, national) sequentially. What stakeholders would influence the uptake of this technology?
27. Do you think healthcare providers would consider information collected and displayed by this device trustworthy? Why or why not?
28. Would you like to see your facility incorporate this device into newborn care? Why or why not?

Feasibility

29. Do you think this device is suitable for your facility? What would need to happen in order to integrate this device successfully at this facility? Please explain.

Probes: For example, staffing availability and skill to use the device, training, complexity of the device, availability of equipment and infrastructure needed for its use, durability and maintenance of device and components, access to spare parts, protocols and guidelines for use, counselling caregivers and informational materials? What could be benefits/drawbacks?

Probes: For example, ease of use during a patient visit, integration into current flow of hospital operations, acceptance by administrators, etc.?

30. Do you have any other comments about this device that we did not talk about?

F. Sibel ANNE investigational device

The next set of question will focus on your experiences with the Sibel ANNE device.

Usability

1. Do you think that healthcare providers in this facility could develop the skills necessary to use this continuous monitoring device? Why or why not?
2. What sort of training did you receive on this device before you began using it?
   Probes: Please describe length and method of training. Who provided training? Was training adequate? What additional training do you wish you had received? What sort of training do you think would be required for healthcare providers in this facility to use this device?
3. Which aspects of using this device were easy to learn? Which aspects were difficult?
   Probes: Did using the device become easier or more difficult over time?
4. If you now feel comfortable using the device, how long did it take you to become comfortable? If not comfortable, why not?
5. What kind of support did you receive during this period? Please explain.
   Probes: From device manufacturers, supervisors, coworkers, etc.?
6. What did you like (if anything) about this device overall? What did you dislike (if anything)? Are there any changes you would make to this device? If so, what are they?
   Probes: For example, overall device setup/interface, ease of use, etc.? What about the different features: chest and limb sensors, hydrogel, mobile application, iPad screen/display, etc.?
7. Did this device make providing care to newborns at this facility easier or more difficult? How so?
   Probes: For example, enable more care, interrupt care, etc.
8. Do you think the device would make care easier or more difficult if you could use the information collected and displayed by this device? How so?
9. Were there any questions you had about this device while you were using it? Please explain.
10. Did caregivers or other hospital staff ask you any questions about this device while you were using it? If so, what did they ask?
11. Are there situations where you think this device should not be used? If so, what are they?

Acceptability

12. How do you think other healthcare providers, like doctors and nurses, would feel about this device?
13. Based on your encounters with caregivers, such as mothers, fathers, mothers-in-law and other family members, how do you think they would feel about this device?
14. How do you think healthcare administrators and decision-makers at local, district and national levels would react to a recommendation to implement this device at this facility?
    Probes: Discuss at each level (local, district, national) sequentially. What stakeholders would influence the uptake of this technology?
15. Do you think healthcare providers would consider information collected and displayed by this device trustworthy? Why or why not?
16. Would you like to see your facility incorporate this device into newborn care? Why or why not?

Feasibility

17. Do you think this device is suitable within your facility? What would need to happen in order to integrate this device successfully at this facility? Please explain.

Probes: For example, staffing availability and skill to use the device, training, complexity of the device, availability of equipment and infrastructure needed for its use, durability and maintenance of device and components, access to spare parts, protocols and guidelines for use, counselling caregivers and informational materials? What could be benefits/drawbacks?

Probes: For example, ease of use during a patient visit, integration into current flow of hospital operations, acceptance by administrators, etc.?

18. Do you have any other comments about this device that we did not talk about?

G. Masimo RAD-97 reference device

The next set of question will focus on your experiences with the Masimo Rad-97 device.

Usability

1. Do you think that healthcare providers in this facility could develop the skills necessary to use this continuous monitoring device? Why or why not?
2. What sort of training did you receive on this device before you began using it?
   Probes: Please describe length and method of training. Who provided training? Was training adequate? What additional training do you wish you had received? What sort of training do you think would be required for healthcare providers in this facility to use this device?
3. Which aspects of using this device were easy to learn? Which aspects were difficult?
   Probes: Did using the device become easier or more difficult over time?
4. If you now feel comfortable using the device, how long did it take you to become comfortable? If not comfortable, why not?
5. What kind of support did you receive during this period? Please explain.
   Probes: From device manufacturers, supervisors, coworkers, etc.?
6. What did you like (if anything) about this device overall? What did you dislike (if anything)? Are there any changes you would make to this device? If so, what are they?
   Probes: For example, overall device setup/interface, ease of use, etc.? What about the different features: Rad-97 device, skin sensor, capnography tube, mobile application, monitor screen/display, etc.?
7. Did this device make providing care to newborns at this facility easier or more difficult? How so?
   Probes: For example, enable more care, interrupt care, etc.
8. Do you think the device would make care easier or more difficult if you could use the information collected and displayed by this device? How so?
9. Were there any questions you had about this device while you were using it? Please explain.
10. Did caregivers or other hospital staff ask you any questions about this device while you were using it? If so, what did they ask?
11. Are there situations where you think this device should not be used? If so, what are they?

Acceptability

12. How do you think other healthcare providers, like doctors and nurses, would feel about this device?
13. Based on your encounters with caregivers, such as mothers, fathers, mothers-in-law and other family members, how do you think they would feel about this device?
14. How do you think healthcare administrators and decision-makers at local, district and national levels would react to a recommendation to implement this device at this facility?
    Probes: Discuss at each level (local, district, national) sequentially. What stakeholders would influence the uptake of this technology?
15. Do you think healthcare providers would consider information collected and displayed by this device trustworthy? Why or why not?
16. Would you like to see your facility incorporate this device into newborn care? Why or why not?

Feasibility

17. Do you think this device is suitable within your facility? What would need to happen in order to integrate this device successfully at this facility? Please explain.<br>*Probes: For example, staffing availability and skill to use the device, training, complexity of the device, availability of equipment and infrastructure needed for its use, durability and maintenance of device and components, access to spare parts, protocols and guidelines for use, counselling caregivers and informational materials? What could be benefits/drawbacks?*<br>*Probes: For example, ease of use during a patient visit, integration into current flow of hospital operations, acceptance by administrators, etc.?*<br>18. Do you have any other comments about this device that we did not talk about?

**H. Closing**
6. Taking into consideration the three monitoring devices we have talked about today, could you rank the device (if any) you think is the best, second best and third choice in your opinion? Please explain why.<br>7. In terms of feasibility, which device (if any) do you think would be the most appropriate device for your healthcare facility and why?<br>8. In terms of acceptability, which device (if any) do you think healthcare providers would like the best and why? Which device (if any) do you think caregivers would prefer and why?<br>9. Do you have any other comments about any of the three devices that we did not talk about?<br>10. Do you have any other comments about newborn monitoring devices or any other comments or concerns overall that we did not get to talk about?
"Thank you for your time and the helpful information you have provided. Your feedback, along with feedback from other people we talk to will be used to recommend solutions for better care."
**Interview end time:** |H|H| : |M|M| *military time*

2.4 In-Depth Interview Guide – Healthcare Provider (HCP) In-Direct Use

**Administrative information**	
**HCP ID number:**	**Sex:** ☐ Female ☐ Male
**Date HCP informed consent form (ICF) signed:** |D|D| - |M|M|M| - |Y|Y|Y|Y|	
**HCP ICF signed prior to any study questions?** ☐ Yes ☐ No	
**Name of research staff who explained the ICF:**	
**Does the HCP agree to be audio recorded?** ☐ Yes ☐ No<br>If Yes, was the interview audio recorded? ☐ Yes ☐ No<br>If No, why was the interview not recorded? ____________________	
**Name of interviewer:**	
**Date of interview:** |D|D| - |M|M|M| - |Y|Y|Y|Y|	
**Location of interview:**<br>☐ **Aga Khan University – Nairobi Hospital**	

☐ **Pumwani Maternity Hospital**
**Interview start time:** |H|H| : |M|M| *military time*
**<u>Instructions for qualitative research staff</u>:**<br>Use this document as a guide to conduct the interviews with the **healthcare providers (HCP) not directly using the devices**.<br>Conduct the interview in the language with which the HCP feels most comfortable.<br>The interview should take place in a quiet place that allows privacy.<br>Please introduce each question separately. The interview must flow as a conversation. If you notice that the HCP is hesitant in answering, does not give an in-depth response, or the response is not satisfactory, please probe or ask follow-up questions, but do NOT prompt any specific answer. Several probes are suggested, and you may also ask follow-up questions that are not listed in this guide but are necessary for the complete expression of the HCP's views.<br>Please **record the interview** using the audio recorder (if HCP consent is provided) and state the ETNA HCP ID number.<br>All comments from the HCP should be recorded/written verbatim.<br>Please cross-check the narratives written with the recorded version as a reference, and correct as necessary.<br>All responses must be kept confidential. Do not discuss or share responses with anyone outside of the ETNA study team.

**Script to initiate the interview**
"Hello, my name is ___________ and I am a researcher with the ETNA project and we are evaluating monitoring devices for newborns. We want to hear from your experiences and learn from your thoughts and feelings. We will keep what you tell us today confidential, which means that nothing you say will be directly linked to you so please feel free to share. If you feel uncomfortable with any questions, let me know and we will skip it. Before we start, do you have any questions for me? Is it ok to begin? Thank you, I will start the audio-recording now."
**R. Demographic information**
10. First, we will start with some questions about yourself, what is your age?<br>11. How many years of education and training have you completed and what is your highest level of education completed? What is your medical background/designation (e.g., doctor, nurse, technician, etc.)?<br>12. How long have you worked as 'doctor/nurse/technician/etc.?

**S. Healthcare provider role**
8. How long have you been employed at this healthcare facility?<br>9. What is your job title and current role here at this facility? How long have you been in this role at this facility?<br>10. What are your responsibilities in this role?<br>*Probes: Please share what a typical day as a healthcare provider would be like for you.*<br>11. Are you involved in patient care? If yes, please explain your patient care responsibilities.

**T. Facility**

10. What are the current constraints to providing care to newborns at this healthcare facility?
    Probes: What makes care more difficult? What would make it easier?
11. How are newborns monitored at this facility? How is this different (if at all) for sick newborns?
12. Does this facility have reliable access to electricity? When was the last electricity outage and how long do they typically last? What happens during power outages at your facility?
    Probes: How do power outages affect patient care? Is there a back-up power supply? If so, what is the process of using the backup power supply and are there any issues around its use (e.g., does it cover all of the equipment needed, any issues in getting permission for its use, fuel prices? Any voltage issues?)
13. Do you have regular access to computers at this facility? If yes, do they work well?
    Probes: Do computers breakdown often? If yes, please describe how the computer breakdowns affect your work as a healthcare provider?
14. Could you describe the technologies that are being used in the delivery and newborn care wards at this facility? What are some concerns you have, or gaps in the technologies available, for maternal and newborn care at this facility?
    Probes: Which healthcare providers use the technologies? What technologies/brands are used? Do healthcare providers use any handheld or portable devices for maternal or newborn care (e.g.,, tablets or Smartphones)? If yes, please describe the technologies and their use.

U. Monitoring devices

1. Are you familiar with the Evaluation of Technologies for Neonates in Africa (ETNA) research study? Are you involved with the study?
   Probes: Are you familiar with the purpose of the study and/or study procedures? Have you previously spoken with any study staff?

We would especially like to learn about your perspectives on continuous monitoring devices.

2. Are continuous monitoring devices used in any capacity at this healthcare facility?
   Probes: If yes, where in the facility? For what purpose? How frequently do you use these devices?
3. Tell me about your experience with continuous monitoring devices. Have you used devices yourself or seen them used?
   Probes: List devices used, then discuss each device sequentially (where, for what purpose?). How frequently have you used these types of devices? Did you find them to be useful? If yes, how so? If no, why not? What sort of training did you receive for the use of these devices?
4. What do you think are some of the benefits (if any) of using continuous monitoring devices? What impacts do you think they could have on routine care at this facility?
5. Do you have any concerns about using continuous monitoring devices? What are the challenges (if any) to using such devices at this facility? Are there any situations you think the use of monitoring devices would not be useful? If so, could you explain with an example?
6. What do you think would be needed to scale up the use of continuous monitoring devices at this facility? What enablers do you think could support this process?
7. Imagine if continuous monitoring devices were scaled up at this facility, how do you think the nurses and doctors that work here would react? How do you think caregivers (mothers, parents, guardians, etc.) would react?

V. EarlySense InSight investigational device

31. Are you familiar with the EarlySense InSight device?
32. Have you ever seen this device before? Have you used it or seen it being used?

Research staff shows EarlySense InSight to HCP and explains how it works.

"The EarlySense InSight is a contact-free physiological monitoring system. The system includes a sensor pad that is placed under the neonate's mattress and can measure pulse, respiratory rate, motion, and sleep status. There is no direct physical contact between the neonate and the sensor pad.

Information from the sensor pad is continuously transmitted to a central display that can be read by hospital staff."

Allow them to touch the InSight device, the mattress pad and the cable that goes between them.

Usability

33. Do you think that healthcare providers in this facility have the skills necessary to use this device? Why or why not?
34. What sort of training do you think would be required for providers in this facility to be able to use this device?
    Probes: What do think the appropriate length of time and method of delivery of training?
35. Which aspects of using this device do you think would be easy to learn? Which aspects would be difficult?
    Probes: What barriers do you anticipate?
36. How do you think using this device would affect providing care to newborns at this facility?
    Probes: Would using this device make provision of care easier or more difficult?
37. What kind of questions do you think caregivers or other hospital staff would have about this device?
38. Are there any changes you would make to this device? If so, what are they?
39. Are there situations where you think this device should not be used? If so, what are they?

Acceptability

40. What do you like (if anything) about this device overall? What do you dislike (if anything)?
    Probes: For example, overall device setup/interface, InSIght device, mattress pad, cable, mobile application, monitor screen/display, etc.?
41. How do you think other healthcare providers, like doctors and nurses, would feel about this device?
42. How do you think caregivers, such as mothers, fathers, mothers-in-law and other family members, would feel about this device?
43. How do you think healthcare administrators and decision-makers at local, district and national levels would react to a recommendation to implement this device at this facility?
    Probes: Discuss at each level (local, district, national) sequentially. What stakeholders would influence the uptake of this technology?
44. Would you consider information collected and displayed by this device trsutworthy? Why or why not?
45. Do you think this device could be useful for monitoring newborns at this facility? Why or why not?
46. Would you like to see your facility incorporate this device into newborn care? Why or why not?

Feasibility

47. Do you think this device is suitable for your facility? What would need to happen in order to integrate this device successfully at this facility? Please explain.
    Probes: For example, staffing availability and skill to use the device, training, complexity of the device, availability of equipment and infrastructure needed for its use, durability and maintenance of device and components, access to spare parts, protocols and guidelines for use, counselling caregivers and informational materials? What could be benefits/drawbacks?
    Probes: For example, ease of use during a patient visit, integration into current flow of hospital operations, acceptance by administrators, etc.?
48. Do you have any other comments about this device that we did not talk about?

W. Sibel ANNE investigational device

1. Are you familiar with the Sibel ANNE device?
2. Have you ever seen this device before? Have you used it/seen it being used?

Research staff show Sibel Advanced Neonatal Epidermal (ANNE) system to HCP and explains how it works:

"The Sibel Advanced Neonatal Epidermal System, referred to as the ANNE system, uses non-invasive sensors to continuously measure and record a neonate's pulse, respiratory rate, level of oxygen in the blood or SpO2, and skin temperature. One sensor is attached to the neonate's chest and the second sensor is applied to the neonate's foot. Information from the sensors is wirelessly transmitted to a monitor or tablet that can be read by hospital staff."

Allow them to touch the ANNE chest and limb sensors, hydrogel, and tablet display fully.

Usability

3. Do you think that healthcare providers in this facility have the skills necessary to use this device? Why or why not?
4. What sort of training do you think would be required for providers in this facility to be able to use this device?
   Probes: What do think the appropriate length of time and method of delivery of training?
5. Which aspects of using this device do you think would be easy to learn? Which aspects would be difficult?
   Probes: What barriers do you anticipate?
6. How do you think using this device would affect providing care to newborns at this facility?
   Probes: Would using this device make provision of care easier or more difficult?
7. What kind of questions do you think caregivers or other hospital staff would have about this device?
8. Are there any changes you would make to this device? If so, what are they?
9. Are there situations where you think this device should not be used? If so, what are they?

Acceptability

10. What do you like (if anything) about this device overall? What do you dislike (if anything)?
    Probes: For example, overall device setup/interface, ANNE chest and limb sensor, hydrogel, tablet, mobile application, monitor screen/display, etc.?
11. How do you think other healthcare providers, like doctors and nurses, would feel about this device?
12. How do you think caregivers, such as mothers, fathers, mothers-in-law and other family members, would feel about this device?
13. How do you think healthcare administrators and decision-makers at local, district and national levels would react to a recommendation to implement this device at this facility?
    Probes: Discuss at each level (local, district, national) sequentially. What stakeholders would influence the uptake of this technology?
14. Would you trust the information collected and displayed by this device? Why or why not?
15. Do you think this device could be useful for monitoring newborns at this facility? Why or why not?
16. Would you like to see your facility incorporate this device into newborn care? Why or why not?

Feasibility

17. Do you think this device is suitable for your facility? What would need to happen in order to integrate this device successfully at this facility? Please explain.
    Probes: For example, staffing availability and skill to use the device, training, complexity of the device, availability of equipment and infrastructure needed for its use, durability and maintenance of device and components, access to spare parts, protocols and guidelines for use, counselling caregivers and informational materials? What could be benefits/drawbacks?
    Probes: For example, ease of use during a patient visit, integration into current flow of hospital operations, acceptance by administrators, etc.?
18. Do you have any other comments about this device that we did not talk about?

**X. Masimo RAD-97 reference device**
1. Are you familiar with the Masimo RAD-97 device?

2. Have you ever seen this device before? Have you used it/seen it being used?

Research staff shows Masimo Rad-97 device to HCP and explains how it works.

"The Masimo Rad-97 is a non-invasive device that measures a neonate's pulse, respiratory rate, and level of oxygen in the blood or SpO2, in a hospital setting. Information is collected through a skin sensor that is applied to the newborn's hand or foot and a cannula tube that is inserted into the neonate's nostrils. The information is then continuously transmitted to a monitor that can be read by hospital staff."

Allow them to touch the Rad-97, skin sensor and capnography tube fully.

Usability

3. Do you think that healthcare providers in this facility have the skills necessary to use this device? Why or why not?
4. What sort of training do you think would be required for providers in this facility to be able to use this device?
   Probes: What do think the appropriate length of time and method of delivery of training?
5. Which aspects of using this device do you think would be easy to learn? Which aspects would be difficult?
   Probes: What barriers do you anticipate?
6. How do you think using this device would affect providing care to newborns at this facility?
   Probes: Would using this device make provision of care easier or more difficult?
7. What kind of questions do you think caregivers or other hospital staff would have about this device?
8. Are there any changes you would make to this device? If so, what are they?
9. Are there situations where you think this device should not be used? If so, what are they?

Acceptability

10. What do you like (if anything) about this device overall? What do you dislike (if anything)?
    Probes: For example, overall device setup/interface, Rad-97 device, skin sensor, capnography tube, mobile application, monitor screen/display, etc.?
11. How do you think other healthcare providers, like doctors and nurses, would feel about this device?
12. How do you think caregivers, such as mothers, fathers, mothers-in-law and other family members, would feel about this device?
13. How do you think healthcare administrators and decision-makers at local, district and national levels would react to a recommendation to implement this device at this facility?
    Probes: Discuss at each level (local, district, national) sequentially. What stakeholders would influence the uptake of this technology?
14. Would you trust the information collected and displayed by this device? Why or why not?
15. Do you think this device could be useful for monitoring newborns at this facility? Why or why not?
16. Would you like to see your facility incorporate this device into newborn care? Why or why not?

Feasibility

17. Do you think this device is suitable for your facility? What would need to happen in order to integrate this device successfully at this facility? Please explain.
    Probes: For example, staffing availability and skill to use the device, training, complexity of the device, availability of equipment and infrastructure needed for its use, durability and maintenance of device and components, access to spare parts, protocols and guidelines for use, counselling caregivers and informational materials? What could be benefits/drawbacks?
    Probes: For example, ease of use during a patient visit, integration into current flow of hospital operations, acceptance by administrators, etc.?
18. Do you have any other comments about this device that we did not talk about?

H. Closing

11. Taking into consideration the monitoring devices we have talked about today, could you rank the device (if any) you think is the best, second best and third choice in your opinion? Please explain why.
12. In terms of feasibility, which device (if any) do you think would be the most appropriate for your healthcare facility and why?
13. In terms of acceptability, which device (if any) do you think healthcare providers would like the best and why? Which device (if any) do you think caregivers would prefer and why?
14. Do you have any other comments about any of the three devices that we did not talk about?
15. Do you have any other comments about newborn monitoring devices or any other comments or concerns overall that we did not get to talk about?

"Thank you for your time and the helpful information you have provided. Your feedback, along with feedback from other people we talk to will be used to recommend solutions for better care."

Interview end time: |H|H| : |M|M| *military time*
