# Supplementary material for: Multiparameter Continuous Physiological Monitoring Technologies in Neonates Among Health Care Providers and Caregivers at a Private Tertiary Hospital in Nairobi, Kenya: Feasibility, Usability, and Acceptability Study
Source: J Med Internet Res. 2021 Oct 28;23(10):e29755. doi: 10.2196/29755 (PMC8587184; doi:10.2196/29755)
Supplement: Multimedia Appendix 3 [file jmir_v23i10e29755_app3.pdf]

S3 File Healthcare Provider (HCP) Direct Observation Guide

A. Administrative information
**HCP ID number:**
**Date HCP informed consent form (ICF) signed:** |D|D| - |M|M| - |Y|Y|Y|Y|
**HCP ICF signed prior to any observation?** ☐ Yes ☐ No<br>If no, please do not make any observations until the ICF has been completed.
**Name of observer:**
**Neonate ID number:**
**Date of observation:** |D|D| - |M|M| - |Y|Y|Y|Y|
**Location of observation:**<br>☐ **Aga Khan University Hospital – Nairobi**<br>☐ **Pumwani Maternity Hospital**
**Observation start time:** |H|H| : |M|M| *military time*<br>*(Time HCP began device preparation)*
**There are three different phases that can be observed and reported in the fields below:**<br>1. Device preparation and initial application: observing HCP prepare and place device on neonate.<br>2. Ongoing device monitoring and troubleshooting: observing HCP perform regular checks of device placement on neonate (and repositioning if necessary) and data quality, including troubleshooting.<br>3. Device disconnection, removal, and cleaning: observing HCP remove device from neonate, clean and store.<br>**Instructions for qualitative research staff:**<br>Use this document as a guide to conduct observations of one HCP during one or more of the phases described above. Indicate in checklist below which phase(s) were included in this observation session.<br>**Use a new form for each HCP**. Two different HCP should not be included on the same form. **Use a new form for each neonate and for each observation session day**. Two different neonates should not be included on the same form. Two different observation session days should not be included on the same form. Multiple observations of the same neonate by the same HCP on the same day can be included on the same form.

Record observations. All observations must be kept confidential. Do not discuss or share observations with anyone outside of the ETNA study team.

**B. Phase(s) observed during this session on the same neonate on the same day (check all that apply)**
☐ Device preparation and initial application<br>☐ Ongoing device monitoring and troubleshooting<br>☐ Device disconnection, removal and cleaning

**C. Which devices did the HCP use during today's observation?**		
EarlySense InSight investigational device	□ Yes	□ No
Sibel ANNE investigational device	□ Yes	□ No
Masimo Rad-97 reference device	□ Yes	□ No

**D. PHASE 1: Device initial application**
**EarlySense InSight investigational device** *(if device not used, skip to next section)*
Application start time: |H|H| : |M|M| military time<br>Application end time: |H|H| : |M|M| military time<br><br>□ Did not complete device preparation and initial application
Please check those steps that you observed. Comments and observations can be made below.<br><br>***Preparation***<br>☐ Remove neonate from bed/bassinet<br>☐ Place pad under neonate's mattress<br>☐ Gently place neonate back on bed/bassinet with chest above middle of pad<br>☐ Attach pad cord to InSight device<br>☐ Confirm InSight device is seen on EarlySense laptop/CDS<br>***Admission***<br>☐ Correct name of admitting nurse selected in EarlySense laptop/CDS<br>☐ Enter PTID into EarlySense laptop/CDS admit patient screen in MRN (ID) box
Please provide comments if HCP <u>did not complete</u> device preparation and initial application.<br>Also, if HCP was not able to complete steps correctly, what did they do instead?

Did you observe HCP have any challenges or difficulties preparing and/or applying device? What were the problems and how were they resolved?

Did HCP require any assistance when preparing and/or applying device?
□ Yes □ No
If yes, who assisted HCP?
If yes, what kind of assistance was required?

Did you observe any risky situations where mistakes could potentially happen, such as times when HCP almost made a mistake? If yes, please explain.

Did HCP make any other comments to you or their colleagues related to preparing and/or applying device? If yes, record comments verbatim and provide context as necessary.

Sibel ANNE investigational device *(if device not used, skip to next section)*

Application start time: |H|H| : |M|M| military time
Application end time: |H|H| : |M|M| military time

□ Did not complete device preparation and initial application

Please check those steps that you observed. Comments and observations can be made below.

Preparation for data collection

- □ ANNE Connect application opened immediately after Sibel iPad unlocked
- □ Participant ID entered to start data collection session
- □ Correct chest and limb sensors selected from within ANNE Connect app

Application of ANNE chest sensor

- □ Open new hydrogel package and apply hydrogel adhesive to chest sensor or neonate's chest, with gentle but firm pressure
- □ Place chest sensor on the torso of the neonate and apply gentle but firm pressure to secure sensor to hydrogel adhesive

Application of ANNE limb sensor

- □ Insert limb sensor into Velcro strap holes Apply LED to bottom of neonate's foot
- □ Apply limb sensor on neonate's foot with LED to bottom of neonate's foot Check that photodiode is aligned with LED
- □ Confirm proper limb sensor placement by checking ANNE Connect application to verify that an error message is not displayed

Confirmation of data collection

- □ Correctly close ANNE Connect application (without disconnecting within Connect app)
- □ Open ANNE Stream application to check quality of vital signs signals

Please provide comments if HCP <u>did not complete</u> device preparation and initial application.
Also, if HCP was not able to complete steps correctly and **in order**, what did they do instead?

Did you observe HCP have any challenges or difficulties preparing and/or applying device? What were the problems and how were they resolved?

Did HCP require any assistance when preparing and/or applying device?
□ Yes □ No
If yes, who assisted HCP?
If yes, what kind of assistance was required?

Did you observe any risky situations where mistakes could potentially happen, such as times when HCP almost made a mistake? If yes, please explain.

Did HCP make any other comments to you or their colleagues related to preparing and/or applying device? If yes, record comments verbatim and provide context as necessary.
**Masimo Rad-97 reference device** *(if device not used, skip to next section)*
Application start time: |H|H| : |M|M| military time<br>Application end time: |H|H| : |M|M| military time<br>□ Did not complete device preparation and initial application
Please check those steps that you observed. Comments and observations can be made below.<br>☐ Power on Rad-97 device<br>☐ Plug in a RD Rainbow SET Series Patient Cable to Patient Cable Port on front of Rad-97 device<br>☐ Plug in new, unused NomoLine Infant Cannula to round NomoLine Capnography Input Connector on front of Rad-97 device<br>☐ Attach RD SET Series SpO2 Disposable Sensor to Patient Cable<br>☐ Apply skin sensor to hand or foot<br>☐ Ensure sensor wrapped securely but not too tightly and ensure correct alignment of light and detector<br>☐ Cover sensor to avoid interference from external light sources (as needed)<br>☐ Insert capnography tubing into nostrils, ensuring that the cannula is not obstructed from collecting CO_2<br>☐ Secure cannula in place using neonate-safe adhesive as needed<br>☐ Ensure good quality (square) capnography waveform and high signal quality (perfusion index or PI) on Rad-97 monitor
Please provide comments if HCP <u>did not complete</u> device preparation and initial application.<br>Also, if HCP was not able to complete steps correctly, what did they do instead?
Did you observe HCP have any challenges or difficulties preparing and/or applying device? What were the problems and how were they resolved?
Did HCP require any assistance when preparing and/or applying device?<br>□ Yes □ No<br>If yes, who assisted HCP?<br>If yes, what kind of assistance was required?
Did you observe any risky situations where mistakes could potentially happen, such as times when HCP almost made a mistake? If yes, please explain.
Did HCP make any other comments to you or their colleagues related to preparing and/or applying device? If yes, record comments verbatim and provide context as necessary.
**E. PHASE 2: Ongoing device monitoring and troubleshooting**
**EarlySense InSight investigational device** *(if device not used, skip to next section)*
Did you observe HCP have any challenges or difficulties with device monitoring and/or troubleshooting? What were the problems and how were they resolved?
Did HCP do any troubleshooting during ongoing monitoring? If so, please describe what the issues were, how the HCP addressed them and an estimate for how long it took.

Issue	Solution	Start Time	End Time

Did HCP require any assistance when monitoring the EarlySense InSIght investigational device?<br>□ Yes □ No<br>If yes, who assisted HCP?<br>If yes, what kind of assistance was required?
Did you observe any risky situations where mistakes could potentially happen, such as times when HCP almost made a mistake? If yes, please explain.
Did HCP make any other comments to you or their colleagues related to device monitoring and/or troubleshooting? If yes, record comments verbatim and provide context as necessary.
**Sibel ANNE investigational device** *(if device not used, skip to next section)*
Did HCP complete the following steps correctly? Please check those steps that you observed. Comments and observations can be made below.<br>☐ Open ANNE Stream application to check quality of vital signs waveforms (lines) and perfusion index (PI).<br>☐ Take corrective measures to address signal quality issues (if needed)?
If signal quality issues needed to be addressed, what corrective measures did they take?
Did you observe HCP have any challenges or difficulties with device monitoring and/or troubleshooting? What were the problems and how were they resolved?
Did HCP do any troubleshooting during ongoing monitoring? If so, please describe what the issues were, how the HCP addressed them and an estimate for how long it took.

Issue	Solution	Start Time	End Time

Did HCP require any assistance when monitoring the Sibel ANNE investigational device?<br>□ Yes □ No<br>If yes, who assisted HCP?<br>If yes, what kind of assistance was required?
Did you observe any risky situations where mistakes could potentially happen, such as times when HCP almost made a mistake? If yes, please explain.
Did HCP make any other comments to you or their colleagues related to device monitoring and/or troubleshooting? If yes, record comments verbatim and provide context as necessary.
**Masimo Rad-97 reference device** *(if device not used, skip to next section)*
Did HCP complete the following steps correctly? Please check those steps that you observed. Comments and observations can be made below.<br>☐ Confirm adequate signal quality (PI) for skin sensor<br>☐ Confirm adequate signal quality (waveform) for capnography tube
If signal quality issues needed to be addressed, what corrective measures did they take?<br>☐ Confirm placement of skin sensor<br>☐ Confirm placement of cannula<br>☐ Confirm connection of Patient Cable to Patient Cable port<br>☐ Confirm connection of Capnography Input Connector<br>☐ Other ____

Did you observe HCP have any challenges or difficulties with device monitoring and/or troubleshooting? What were the problems and how were they resolved?

Did HCP do any troubleshooting during ongoing monitoring? If so, please describe what the issues were, how the HCP addressed them and an estimate for how long it took.

Issue	Solution	Start Time	End Time

Did HCP require any assistance when monitoring the Masimo Rad-97 reference device?
□ Yes □ No
If yes, who assisted HCP?
If yes, what kind of assistance was required?

Did you observe any risky situations where mistakes could potentially happen, such as times when HCP almost made a mistake? If yes, please explain.

Did HCP make any other comments to you or their colleagues related to device monitoring and/or troubleshooting? If yes, record comments verbatim and provide context as necessary.

F. PHASE 3: Device disconnection, removal, and cleaning

EarlySense InSight investigational device *(if device not used, skip to next section)*

Did HCP discharge neonate from EarlySense laptop/CDS correctly? If not, what did HCP do instead?

Did you observe HCP have any challenges or difficulties with device disconnection, removal, and/or cleaning? What were the problems and how were they resolved?

Did the HCP require any assistance with device disconnection, removal, and/or cleaning?
□ Yes □ No
If yes, who assisted the HCP?
If yes, what kind of assistance was required?

Did you observe any risky situations where mistakes could potentially happen, such as times when HCP almost made a mistake? If yes, please explain.

Did HCP make any other comments to you or their colleagues related to device disconnection, removal, and/or cleaning? If yes, record comments verbatim and provide context as necessary.

Sibel ANNE investigational device *(if device not used, skip to next section)*

Please check those steps that you observed. Comments and observations can be made below.

- □ Disconnect chest and limb sensors from Devices tab of ANNE Stream application
- □ Close ANNE Stream application
- □ Close ANNE Sync application by swiping up on application
- □ Re-open ANNE Connect application
- □ Disconnect limb sensor first
- □ Disconnect chest sensor
- □ End session by selecting “End Session” button from ANNE Connect application
- □ Sanitize hands according to study site infection control policy
- □ Remove chest sensor by gently pulling off, away from the neonate, on one corner
- □ Gently remove any residual adhesive using a saline cleaning wipe
- □ Unfasten Velcro button from strap and remove limb sensor

- ☐ Dispose of used Velcro strap
- ☐ Clean chest and limb sensors, wipe both sides
- ☐ Dispose used cleaning wipe

If HCP was not able to complete steps correctly and in order, what did they do instead?

Did you observe HCP have any challenges or difficulties with device disconnection, removal, and/or cleaning? What were the problems and how were they resolved?

Did the HCP require any assistance with device disconnection, removal, and/or cleaning?
□ Yes □ No
If yes, who assisted the HCP?
If yes, what kind of assistance was required?

Did you observe any risky situations where mistakes could potentially happen, such as times when HCP almost made a mistake? If yes, please explain.

Did HCP make any other comments to you or their colleagues related to device disconnection, removal, and/or cleaning? If yes, record comments verbatim and provide context as necessary.

Masimo Rad-97 reference device *(if device not used, skip to next section)*

Did HCP complete the following steps correctly? Please check those steps that you observed. Comments and observations can be made below.

- ☐ Remove adhesive (if present) and capnography tube gently from neonate
- ☐ Carefully remove skin sensor from neonate
- ☐ Dispose of single use capnography tube and disposable skin sensor
- ☐ Unplug capnography tube and patient cable from Rad-97
- ☐ Unplug skin sensor from patient cable
- ☐ Turn off Rad-97

If HCP was not able to complete steps correctly, what did they do instead?

Did you observe HCP have any challenges or difficulties with device disconnection, removal, and/or cleaning? What were the problems and how were they resolved?

Did the HCP require any assistance with device disconnection, removal, and/or cleaning?
□ Yes □ No
If yes, who assisted the HCP?
If yes, what kind of assistance was required?

Did you observe any risky situations where mistakes could potentially happen, such as times when HCP almost made a mistake? If yes, please explain.

Did HCP make any other comments to you or their colleagues related to device disconnection, removal, and/or cleaning? If yes, record comments verbatim and provide context as necessary.

Please note below any further comments that may have not already been covered in above sections. In particular, if you have any observations comparing the HCP's use of the different devices, if applicable.
