# Supplementary material for: Multiparameter Continuous Physiological Monitoring Technologies in Neonates Among Health Care Providers and Caregivers at a Private Tertiary Hospital in Nairobi, Kenya: Feasibility, Usability, and Acceptability Study
Source: J Med Internet Res. 2021 Oct 28;23(10):e29755. doi: 10.2196/29755 (PMC8587184; doi:10.2196/29755)
Supplement: Multimedia Appendix 4 [file jmir_v23i10e29755_app4.docx]

**S4 File Coding tree**

| **Nodes** | **Sub-nodes** | **Description** |
| --- | --- | --- |
| 1. Social-demographics information | 1. Age | Age of participant |
|  | 1. Job title | Job title and current role at the facility |
|  | 1. Employment duration at facility | Duration of employment at the healthcare facility |
|  | 1. Work experience | Duration worked as a physician, nurse, technician, etc. |
|  | 1. Education | Years of education and training completed, highest level of education completed, medical background/designation (e.g., physician, nurse, technician, etc.) |
|  | 1. Healthcare provider role | Responsibilities, patient care responsibilities |
| 1. Health system factors | 1. Current constraints | Description of the current constraints to providing care to newborns at the healthcare facility. Factors that make care more difficult or easy |
|  | 1. Monitoring of newborns at the facility | Methods of newborn monitoring at the facility. How it is different (if at all) for sick newborns |
|  | 1. Access to electricity | Description of whether the facility have reliable access to electricity. The last electricity outage and how long do they typically last. What happens during power outages at the facility. How do power outages affect patient care. A back-up power supply. The process of using the backup power supply and any issues around its use (e.g., does it cover all of the equipment needed, any issues in getting permission for its use, fuel prices. Any voltage issues. |
|  | 1. Access to computers | Description of whether they have regular access to computers at this facility Whether they work well. Computers breakdown. Ways in which the computer breakdowns affect ones work as a healthcare provider |
|  | 1. Technologies used in delivery and newborn unit | Description of the technologies that are being used in the delivery and newborn care wards at this facility. Concerns or gaps in the technologies available, for maternal and newborn care at the facility. Type of healthcare providers who use the technologies. Technologies/ brands used. Whether the healthcare providers use |
| 1. Monitoring devices | 1. Familiarity with role and responsibilities with ETNA | Role with the ETNA research study and any ETNA-related responsibilities |
|  | 1. Use of continuous monitoring devices | Use of continuous monitoring devices or seen them used. Experience with continuous monitoring devices. List of devices used, how frequently one has used the types of devices. usefulness. Training received for the use of the devices. |
|  | 1. Experience with continuous monitoring devices | Description of whether continuous monitoring devices apart from the ETNA devices are used at the healthcare facility. If so, where in the facility, their purpose and frequency of use. |
|  | 1. Benefits | Benefits of using continuous monitoring devices and impacts on routine care at the facility |
|  | 1. Concerns | Any concerns about using continuous monitoring devices. Challenges to using such devices at this facility. Any situations in which the use of monitoring devices would not be useful. |
|  | 1. Need for scale up | What would be needed to scale up the use of continuous monitoring devices at the facility. Enablers that could support the process. |
|  | 1. Reaction on use of monitoring devices | Reaction of the nurses and physicians if use of continuous monitoring devices were scaled up at the facility. Reaction of caregivers (mothers, parents, guardians, etc.) |
|  | 1. Training | Any mention around training and training needs for monitoring devices in general |
| 1. EarlySense investigational device | 1. Familiarity with device | Previous experience with the device |
|  | 1. Usability | Discussions around device usability, likes and dislikes about the device, situations where the device should not be used |
|  | 1. Acceptability | Feelings of healthcare providers, administrators and caregivers about the device, whether they trusted results and if device should be incorporated |
|  | 1. Feasibility | Discussions whether the device would be suitable within their health setting |
| 1. Sibel investigational device | 1. Familiarity with device | Previous experience with the device |
|  | 1. Usability | Discussions around device usability, likes and dislikes about the device, situations where the device should not be used |
|  | 1. Acceptability | Feelings of healthcare providers, administrators and caregivers about the device, whether they trusted results and if device should be incorporated |
|  | 1. Feasibility | Discussions whether the device would be suitable within their health setting |
| 1. Masimo RAD-97 reference device | 1. Familiarity with device | Previous experience with the device |
|  | 1. Usability | Discussions around device usability, likes and dislikes about the device, situations where the device should not be used |
|  | 1. Acceptability | Feelings of healthcare providers, administrators and caregivers about the device, whether they trusted results and if device should be incorporated |
|  | 1. Feasibility | Discussions whether the device would be suitable within their health setting |
| 1. Closing | 1. Rank device | Rank of the device as the best, second best and third choice |
|  | 1. Feasibility – most appropriate device | In terms of feasibility, device (if any) that would be the most appropriate device for the healthcare facility |
|  | 1. Acceptability – most preferred device | In terms of acceptability, device (if any) that the healthcare providers and caregiver would like the best. |
|  | 1. Other comments about the devices | Any other comments about three ETNA study devices in general |
|  | 1. Any other comments about newborn monitoring devices or any other comments or concerns | Any other comments about newborn monitoring devices or any other comments or concerns |
