# Supplementary material for: Multiparameter Continuous Physiological Monitoring Technologies in Neonates Among Health Care Providers and Caregivers at a Private Tertiary Hospital in Nairobi, Kenya: Feasibility, Usability, and Acceptability Study
Source: J Med Internet Res. 2021 Oct 28;23(10):e29755. doi: 10.2196/29755 (PMC8587184; doi:10.2196/29755)
Supplement: Multimedia Appendix 5 [file jmir_v23i10e29755_app5.docx]

**S5 Table Feasibility, usability and acceptability factors of investigational and reference technologies with illustrative quotes**

|  | **Investigational technologies** | | **Reference technology** | |
| --- | --- | --- | --- | --- |
|  | **Factor** | **Illustrative quotes** | **Factor** | **Illustrative quotes** |
| **Feasibility**  **factors** | + Easy to train for use  (11/17 HCP and HCA) | EarlySense: “It is easy to use you only need very minimal training required, just placing it under the mattress and it monitors the baby…” HCP-D 2  Sibel: “requires like an on-job training like maybe a few hours because like I said it, is not something that is totally new of from what is being used. You have to tie somewhere, on the maybe big toe or finger and the other one you have to attach it somewhere around the chest so... [training is needed on] the practical bit of it that just requires the healthcare provider to like attach it because is something that you are already used to.” HCP-I 8 | - Longer training due to complexity (3/17 HCP and HCA) | “Probably, this one might be a bit longer. It might need a bit longer time to be trained because of the connection and the power and all that –the complexity of it.” HCP-I 3  “I can see from the device we have ports, so the nurses and doctors will need to be trained on how to insert on the various devices in these ports and read and interpret results.” HCP-I 2 |
|  | - Requirement of ancillary equipment (2/17 HCP and HCA) | Sibel: “…We are using one iPad per set of devices like the chest and the lung is one device…if in the actualization I would need one monitor for each device that would mean to have all those monitors, all those tablets” HCP-D 2 | + Standalone unit  (3/17 HCP and HCA) | “I think RAD-97 would be the most appropriate. Because its one time. I mean, you don’t necessarily need another device to monitor, unlike Sibel where you need a laptop. This one (EarlySense), you attach and the results are displayed on a screen. In terms of feasibility, I would go for the implementation of RAD-97 first before these others” HCA-2 |
|  | - WiFi requirements (6/17 HCP and HCA) | EarlySense: “Because of the time synchronization… It takes time; you need to do it in presence of a WiFi, which sometimes may be low” HCP-D 2 | + Does not require WiFi (2/17 HCP and HCA) | “Aah what I like about it is that it has like I don’t think it requires those WiFi things yeah… so it can be used anywhere any part of the country.” HCP-1 8 |
|  | - Concerns about integration with existing equipment (5/17 HCP and HCA) | EarlySense: “…we would want all the information in one place and not this one here and the other on the other side… so that clinicians are not looking for information in two or three different places…Would I be able to get that baby’s information into that cardiac monitor? And if it is a no, then I wouldn’t buy it because it would mean that nurses have opened a tablet here and there is a cardiac monitor elsewhere. That doesn’t work well for clinical staff.” HCA 3 |  |  |
| **Usability factors** | + Ease of use and useful for care (20/27 CG, HCP and HCA) | EarlySense: “you just put the pad under the mattress and it gives you the results and it doesn’t need a lot of *eeeh*, what do we call it, it doesn’t not need a lot of positioning and repositioning as long as it is under the mattress of the baby it is able to give you the results” HCP-D 2  EarlySense: “is quite simple than our normal standard monitoring device here, so I'll say it looks easier to use” HCP-I 7  Sibel: “the amount of functions that it is able to do, it even have more functions than our current cardiac monitor so that’s a plus that we are having less of manipulation to the baby in terms of attachment but we are having much results, you can see more, you can see more just heart rate, respirations we can see movement, we can see temperature so there are additional things that you can see from it.” HCP-D 1  EarlySense: “it is able to, to, to read all the parameters, as you've said, the, the motion…At a glance, you're able to know all your readings…You're actually able to monitor from, at a glance, you don't need to worry” CG 9  Sibel: “I could see the readings on her iPad, I could see the oxygen, it was on the right levels” CG 2 | + Useful for care  (4/27 CG, HCP and HCA) | “This is a machine that shows several things that you would actually get on a bigger machine. On the bigger cardiac monitor.” HCA 1  “It's just that it's one machine that contains all those things? (Laughs)…Yeah. So that's the good part of it, in that you can be able to give your care very fast, in that you can detect it, it can detect so fast if there's any deviation, if it's a continuous monitoring.” CG 1 |
|  | + wireless features improve work efficiency (13/27 CG, HCP and HCA) | EarlySense: “if it helps in monitoring a child from far, it will really help…It will help you (the nurse) detect if something is wrong faster so that by the time you get to the bay, you already know what you want to do.” CG 1  EarlySense: “The screen that you are using to display the results, you can put it at the nursing station... So, you are able to pick what is happening: you are able to see that this baby is okay, this one is comfortable, sleeping, this one is about it fall so you are able to go and check. So you can monitor many babies at the same time. It will reduce workload of you going to, for example you moving from nursing station to go and check if that baby is okay.” HCP-D 2  EarlySense: “…the coding is remote. It doesn't interfere with routine care. You don't have to unplug. Like sometimes, I have to remove certain wires to be able to examine a baby properly. So, the fact that it leaves the baby unencumbered with all those things I think is a huge advantage.” HCA-2  Sibel: “In most cases, especially with a newborn, and even with the deaths that you find, or the prolonged sickness is at times you're not able to manage temperature. One, you could be in a facility where babies are so many, so the service provider they're overwhelmed, but if there are such devices that they're able to relay information faster, that means so many babies at least can be observed comfortably, so you save lives.” CG 9 |  |  |
|  | - Monitoring disruptions when infants are restless or off the mattress  (5/17 HCP and HCA) | For the EarlySense device only: “But when the baby does not lay all the time... What happens to that baby when it is being carried? That is also an important aspect of bonding with the baby. That would be a bit of a deterring factor when you want those moments… It needs the baby laying down for it to deliver output.” HCA 4 |  |  |
|  | - Concern about appropriate sizing for preterm and LBW infant (7/27 CG, HCP and HCA) | EarlySense: “You can imagine a very tiny baby, under that big board. [I] am not sure it is going to get everything we are looking for...I don’t know if the consultation is at the centre or at the peripheries [of the board]. Where would I place the baby?” HCP-I 5  Sibel: “For the baby’s chest, some of them like the preterms…I don’t know whether it would be so big and then if it is big, then it is not connecting well.” HCA 3 |  |  |
|  | +/- Small size and portability (8/17 HCP and HCA) | EarlySense: “I think it is a fairly good device, the fact that is portable.” HCP-I 2  Sibel: “to make sure that these things aren’t lost by staff. They don’t just put them anywhere. We might buy them, but at the end of the year, they might all be lost. Because, you know, something which is attached and something which is a little bit big might be better. So, these ones are smaller and they might be really misplaced.” HCA 1 | +/- Small size and portability (6/17 HCP and HCA) | “First of all, it is portable, you can move with it wherever you want. Even if you are transporting a baby you can just hold it and go with it. And then, it doesn’t occupy big spaces. So, I think that it is a good machine.” HCA 1  “As long as it does not get lost you know this is something portable so you need someone accountable” HCP-I 9 |
|  | +/- Ability for infection control  (7/17 HCP and HCA) | EarlySense: “Even if it is going under the mattress everything has to be disinfected as long as… because we never know what is…micro-organisms also grow under there.” HCA 1  Sibel: “The only issue that on my side which is also a challenge which I would say is also how to clean it. Because it is not disposable” HCA 1  Sibel: “at times you are trying to do a procedure that will be maybe bloody that can get stained.. I have seen this is like plastic so cleaning it will be very easy” HCP-I 8 |  |  |
| **Acceptability factors** | + Perceptions of infant comfort (16/27 CG, HCA, HCP) | EarlySense: “It doesn't interfere in any way with the baby. The baby is sleeping they can just sleep, you know? ...It's painless while it's measuring.” CG 7  Sibel: “it’s going to be on nowhere in, nothing to prick the baby…the baby didn’t seem uncomfortable with it, I was there, so I thought…it didn’t cause the baby any discomfort, so I though the use was okay” CG 2 | - Perceptions of infant discomfort (12/27 CG, HCA, HCP) | “I didn't like it so much…It looked a bit uncomfortable, and you could tell she was trying to, you know, move it, and move it out of the way… I didn't like the tube on the nose.” CG 5  “capnography, sometimes it is a bit uncomfortable for the baby. Yeah and most of the mothers, actually 80% of the mother’s declines are not comfortable the way it is attached to the nose of the baby some think it is uncomfortable for the baby” HCP-D 2 |
|  | + Simple and less intimidating (5/10 CG) | Sibel: “It's, it's simple. I mean, you know sometimes too many wires tend to shock someone. You might feel that the baby is in danger or something. So when they place such a thing, even the, the parent or the guardian feels a bit comfortable.” CG 4 | - Wire and tubing associated with critical care (12/27 CG, HCA, HCP) | “For some, they can get frightened…For some, while they are happy that the baby is being monitored, it also creates a little more of anxiety because even they can see things and unfortunately, they are seeing things that they cannot interpret. That can also create a little anxiety” HCA 3  ““I freaked out because having been in the same situation for the past one week and you could see the struggle the baby was having. The first question that came into my mind is the baby going to struggle again breathing using those tubing on the nose…” CG 3  “the wires tend to make you feel like something is really wrong, something is not going right. So when it's just simple, (phone ringing) uh, it makes it much easier, even for the parent, there's a lot of comfort, I mean, the baby's okay. I mean, so when you put too much piping and all that, it sorts of creates a, no, anxiety that the baby's not doing really well.” CG 4  “The fact that we are putting it in the baby’s nostrils, that uncomfortable feeling because even giving oxygen to the neo-nates it is stressing. If the baby is well, do we have to stress the baby more?” HCP-I 10 |
|  | - Concerns about side effects of electrical fields or wireless connection with new technologies (15/27 CG, HCA, HCP) | EarlySense: “okay, they're not using wires, so what are they using? Is there radiation, you know, that can harm my child. Yeah, and I asked, of course, we asked about that, and we were told no, they're safe. So for me it was, like, okay, they don't use wires, so what do they use?” CG 5  Sibel: “yes some have had questions if the device has rays which can be exposed to the baby and be a risk factor to cancer, so we have to reassure them that it doesn’t have, okay whoever it doesn’t have any effect on the baby, yeah” HCP-D 2  Sibel: ““Now, you are not seeing any like wires moving from that device to…the screen next to the baby so they (parents) want to know how that information is being passed. Yeah, of course it will raise some eyebrows and they will wonder yeah how that Bluetooth is working … I…am also wondering how it working with that Bluetooth thing and how is being conducted so…personally I will go with the old version” HCP-I 8 | + Brand or device familiarity  (8/17 HCP and HCA) | “It has been around for a while so reliability has been proven. Especially for the Spo2. I know it to be quite reliable and great to use under the right conditions… The Masimo already is a standard; it has been widely used. So, if I don't have to use an experimental item, if I can go for the gold standard then why not? …You tend to go with what is known. you know? If it is a brand or an equipment that has been used for some time and it is okay, yeah we tend to go with that.” HCA 3  “It is a device that has been used in the past, and its still being used all over the world. It’s a no brainer. It’s like going and asking someone ‘should you drive a Mercedes’ it’s a known brand.” HCP-I 1 |

*CG: caregivers; HCP-I: healthcare providers – indirect user; HCP-D: healthcare providers – direct user; HCA: healthcare administrators*
