# Supplementary material for: Multiparameter Continuous Physiological Monitoring Technologies in Neonates Among Health Care Providers and Caregivers at a Private Tertiary Hospital in Nairobi, Kenya: Feasibility, Usability, and Acceptability Study
Source: J Med Internet Res. 2021 Oct 28;23(10):e29755. doi: 10.2196/29755 (PMC8587184; doi:10.2196/29755)
Supplement: Multimedia Appendix 6 [file jmir_v23i10e29755_app6.docx]

**S6 Table: Themes by technology and study participant group**

|  | **Caregivers** | **Direct healthcare providers** | **Indirect healthcare providers** | **Healthcare administrators** |
| --- | --- | --- | --- | --- |
| **EARLYSENSE investigational technology** | | | | |
| Advantages | - No direct skin contact - Non-invasive and comfortable for infant - Simple and less intimidating - Reports important information and easy to read | - No direct skin contact - Non-invasive and comfortable for infant - Easy to use - Remote monitoring for multiple infants - Easy to train for use | - No direct skin contact - Non-invasive and comfortable for infant - Easy to use - Remote monitoring for multiple infants - Easy to train for use | - Non-invasive and comfortable for infant - Durable |
| Concerns | - Side effects of electrical fields or wireless connection | - Time and WiFi requirements - Monitoring disruptions when infants are restless or off the mattress | - Sanitation and infection control - Side effects of electrical fields or wireless connection - Sizing for preterm and low birthweight infants - Monitoring disruptions when infants are restless or off the mattress | - Sanitation and infection control - Monitoring disruptions when infants are restless or off the mattress - Poor integration with existing equipment |
| **SIBEL investigational technology** | | | | |
| Advantages | - Wireless - Non-invasive and comfortable for infant - Simple and less intimidating - Reports important information and easy to read | - Wireless - Easy to use - Comprehensive functions - Easy to train for use | - Wireless - Easy to use - Remote monitoring for multiple infants - Small and portable - Accommodates infant movement - Ease for cleaning - Easy to train for use | - Wireless - Non-invasive and comfortable for infant - Accommodates infant movement |
| Concerns | - Sanitation and infection control - Side effects of electrical fields or wireless connection - Infant skin irritation - Placement of chest sensor | - Side effects of electrical fields or wireless connection - Infant skin irritation - Requires supporting equipment (screens) | - Side effects of electrical fields or wireless connection - Infant skin irritation - Sizing for preterm and low birthweight infants - Placement of chest sensor - Preventing misplacement or theft | - Sanitation and infection control - Infant skin irritation - Sizing for preterm and low birthweight infants - Additional responsibilities and workload |
| **MASIMO RAD-97 reference technology** | | | | |
| Advantages | - Reports important information - Familiarity and trusted accuracy | - Familiarity and trusted accuracy | - Well known brand - Familiarity and trusted accuracy - Small and portable - Standalone unit | - Well known brand - Familiarity and trusted accuracy - Small and portable |
| Concerns | - Infant discomfort - Wires and tubing associated with critical care | - Infant discomfort - Wires and tubing associated with critical care | - Infant discomfort - Wires and tubing associated with critical care - Interferes with infant care and movement - Preventing misplacement or theft - Longer training requirements | - Wires and tubing associated with critical care - Interferes with infant care and movement |
